# Exosomal circWDR62 promotes temozolomide resistance and malignant progression through regulation of the miR-370-3p/MGMT axis in glioma

**DOI:** 10.1038/s41419-022-05056-5

**Published:** 2022-07-11

**Authors:** Xiuchao Geng, Yuhao Zhang, Xiaomeng Lin, Zhaomu Zeng, Jun Hu, Liangchao Hao, Jianglong Xu, Xinjuan Wang, Hong Wang, Qiang Li

**Affiliations:** 1 Taizhou Central Hosiptal (Taizhou University Hospital), Taizhou University, Taizhou, 318000 Zhejiang China; 2 School of Medicine, Taizhou University, Taizhou, 318000 Zhejiang China; 3 Department of Neurosurgery, Zhejiang Provincial People’s Hospital, Affiliated to Hangzhou Medical College, Hangzhou, 310000 Zhejiang China; 4 Key Laboratory of Precise Diagnosis and Treatment of Glioma in Hebei Provience, Baoding, 071000 Hebei China; 5 Departments of Breast Surgery, Affiliated Hospital of Hebei University, Baoding, 071000 Hebei China; 6 Department of Neurosurgery, Affiliated Hospital of Hebei University, Baoding, 071000 Hebei China; 7 Faculty of Integrated Traditional Chinese and Western Medicine, Hebei University of Chinese Medicine, Shijiazhuang, 050091 Hebei China; 8 Department of Neurosurgery, Affiliated Hospital of Chengde medical University, Chengde, 067000 Hebei China; 9 Department of Plastic Surgery, Shaoxing People’s Hospital, Shaoxing, 312000 Zhejiang China

**Keywords:** CNS cancer, Cancer epigenetics

## Abstract

Exosome-mediated delivery of circular RNAs (circRNAs) is implicated in cancer progression. However, the role of exosomal circRNAs in the chemotherapy resistance of tumours remains poorly understood. Here we identified a novel circRNA, circWDR62. It was found that circWDR62 expression was upregulated in TMZ-resistant glioma cells and TMZ-resistant glioma cell-derived exosomes compared with their controls by using high-throughput microarray analysis and quantitative real-time polymerase chain reaction, and high circWDR62 expression was associated with poor prognosis of glioma. Functionally, downregulation of circWDR62 expression could significantly inhibit the TMZ resistance and malignant progression of glioma. Further mechanistic studies showed that circWDR62 plays a role by sponging miR-370-3p as a competing endogenous RNA. Rescue experiments confirmed that MGMT is the downstream target of the circWDR62/miR-370-3p axis in glioma. In addition, circWDR62 could be transported between TMZ-resistant and TMZ-sensitive glioma cells via exosomes. Exosomal circWDR62 from TMZ-resistant cells conferred TMZ resistance in recipient sensitive cells while also enhancing the proliferation, migration and invasion of these cells. A series of clinical and in vivo trials corroborated that exosomal circWDR62 could promote TMZ chemoresistance and malignant progression of glioma. Our results demonstrate for the first time that exosome-mediated delivery of circWDR62 can promote TMZ resistance and malignant progression via targeting of the miR-370-3p/MGMT axis in vitro and in vivo in glioma, providing a new therapeutic strategy. Moreover, exosomal circWDR62 in human serum may serve as a promising therapeutic target and prognostic marker for glioma therapy.

## Introduction

Glioma is the most common primary malignant tumour in the central nervous system, and the prognosis of glioblastoma (GBM) is still very poor, with a high recurrence rate and a median survival time of only 14.6 months [1]. Temozolomide (TMZ) is the international standard chemotherapy drug for GBM. TMZ resistance and tumour recurrence have become the main obstacles in the clinical treatment of GBM patients [2]. At present, the underlying mechanism leading to TMZ resistance in glioma remains unclear.

Circular RNAs (circRNAs) are single-stranded noncoding RNAs (ncRNAs) with a covalent closed-loop structure. circRNAs do not have a polyadenylate tail or 5' and 3' termini and can thus resist degradation by ribonuclease R (RNase R) [3, 4]. At present, many studies have shown that circRNAs are abnormally expressed in a variety of cancers, including glioma [5, 6], and are closely related to tumour proliferation, migration, and invasion [7, 8]. Recently, it was reported that circRNAs also play an important regulatory role in mediating chemotherapy resistance in cancer [9, 10]. For instance, studies have shown that the expression of circRNA-SORE is upregulated in sorafenib-resistant hepatocellular carcinoma cells and that circRNA-SORE can activate the Wnt/β-catenin signalling pathway by sponging miR-103a-2-5p and miR-660-3p, promoting sorafenib resistance in hepatocellular carcinoma [11]. The main biological function of circRNAs is the regulation of gene expression at the transcriptional or posttranscriptional level [12]. circRNAs can play a biological role by regulating the expression of parent genes [13], acting as miRNA sponges [14], acting as RNA-binding protein (RBP) sponges [15] or protein scaffolds [16], regulating translation [17], and influencing the translation of precursors into proteins or polypeptides [18]. circRNAs serve as competing endogenous RNAs (ceRNAs) are widely recognized to be involved in malignant progression [19]. However, to date, the role of circWDR62 in TMZ resistance in glioma has not been fully explored.

Exosomes are extracellular phospholipid bilayer vesicles with a diameter of 30–150 nm and are secreted by living cells [20]. Exosomes can be used as carriers to encapsulate and transfer functional molecules, including proteins, lipids, messenger RNA (mRNA), and ncRNA [21]. Studies have confirmed that as important vehicles for intercellular communication, exosomes can transfer cargo from derived cells to target cells and thus play an important role in tumour progression and chemotherapy resistance [22]. For example, studies have shown that multidrug-resistant cells deliver miR-32-5p to sensitive receptor cells through exosomes, activate the PI3K/AKT pathway, and further induce multidrug resistance in liver cancer by regulating angiogenesis and epithelial-mesenchymal transition (EMT) [23]. In addition, exosomes can deliver lncRNA SBF2-AS1 to spread TMZ resistance by regulating the miR-151a-3p/XRCC4 axis in glioma cells [24]. However, studies on exosomal circWDR62-mediated tumour chemotherapy resistance are still lacking, and the specific effects and mechanisms are still unclear. More in-depth and extensive studies are urgently needed.

This study aims to explore the biological function and potential mechanism of the novel circRNA circWDR62 in glioma TMZ resistance in vitro and in vivo and whether exosomal circWDR62 is involved in the development of TMZ resistance in glioma. Our findings demonstrate that circWDR62 can be incorporated into exosomes and that exosomal circWDR62 might function as a miR-370-3p sponge to regulate MGMT, promoting the TMZ resistance and malignant development of glioma in vitro and in vivo. Our findings highlight a novel direction underlying the development of TMZ resistance in glioma and provide a new potential target for the treatment of TMZ-resistant glioma.

## Results

### TMZ-resistant glioma cells were successfully established

To explore the effect and mechanism of circRNAs influencing TMZ chemotherapy resistance in glioma cells, we first successfully established stable TMZ-resistant glioma cell lines by gradient induction (Additional file: Fig. S1A). The CCK-8 assay revealed that the IC50 of TMZ in U343-R cells (1797 ± 78.78 μM) was higher than that in U343 cells (67.74 ± 6.92 μM) (Additional file: Fig. S1B), resistance index (RI) = 26.53. Then, we further investigated the response of U343-R and U343 cells to TMZ in vivo. After tumour formation, the tumour size of the U343 group was significantly reduced following TMZ treatment, while the decrease in the tumour size in the U343-R group was not statistically significant. In addition, the tumour weight and volume of the U343-R group were higher than those of the U343 group, and the body weight of the mice showed no significant increase (Additional file: Fig. S1C–F). Both in vivo and in vitro experiments proved that TMZ-resistant glioma cells were successfully established. U251-R cells were also generated by the same method.

### circWDR62 expression is upregulated in TMZ-resistant glioma and is significantly correlated with poor prognosis

**Fig. 1:**
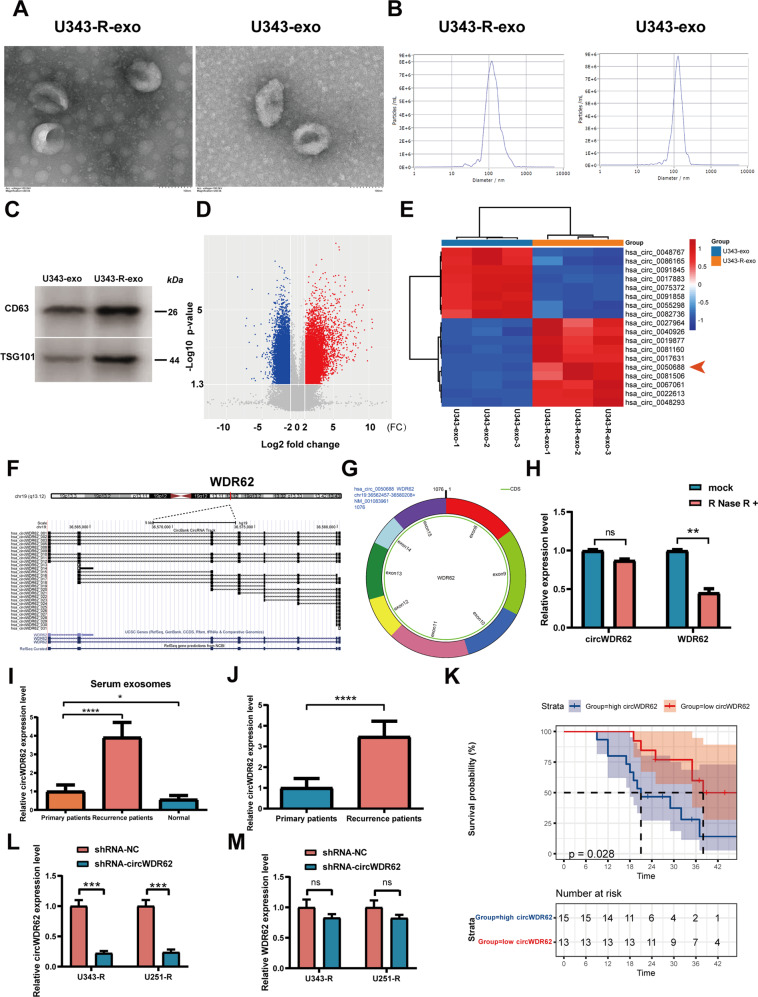
circWDR62 expression is upregulated in TMZ-resistant glioma and expression is significantly correlated with poor prognosis. **A** Representative electron micrograph images of exosomes secreted by U343-R and U343 cells. **B** NTA analysis of the concentration and size distribution of exosomes secreted by U343-R and U343 cells. **C** WB analysis showing the expression levels of the exosome markers CD63 and TSG101 in exosomes secreted by U343-R and U343 cells. **D** Volcano plot showing thousands of differentially expressed circRNAs in U343-R-exos compared to U343-exos. **E** Heatmap showing differentially expressed circRNAs in U343-R-exos vs. U343-exos. hsa_circ_0050688 was one of the most upregulated circRNAs (red arrow). **F** The structure of hsa_circ_0050688 and the genomic position and chromosomal localization of the host gene WDR62 according to the UCSC genome browser (http://genome.ucsc.edu/). **G** Schematic representation of circWDR62. **H** qRT–PCR was conducted to determine the relative expression of circWDR62 and WDR62 upon RNase R treatment. **I** qRT–PCR was conducted to determine the expression of circWDR62 within serum exosomes in patients with primary tumours and recurrent tumours after TMZ-based chemotherapy and normal controls. **J** qRT–PCR was conducted to determine the expression of circWDR62 within tissues in patients with primary tumours and recurrent tumours. **K** Kaplan–Meier survival analysis of patients with recurrent glioma was conducted according to the expression of circWDR62 using the log-rank test. **L** qRT–PCR assay was conducted to verify the expression of circWDR62 in U343-R and U251-R cells after with shRNA-circWDR62. **M** The mRNA expression of WDR62 after transfection of shRNA-circWDR62.

To explore the expression of circRNAs in TMZ-resistant glioma cells-derived exosomes, we first isolated exosomes derived from glioma cells. TEM confirmed that the exosomes secreted by U343-R and U343 cells were round (Fig. 1A). NTA analysis revealed that the concentrations of U343-R cell-derived exosomes (U343-R-exos) and U343 cell-derived exosomes (U343-exos) were ~12.0 × 10^7^ and 8.6 × 10^7^ particles per ml, respectively, and that U343-R cells had a higher concentration of exosomes than U343 cells. The diameter of most particles was within 50–250 nm; the median diameter of U343-R-exos was 142.4 nm, and that of U343-exos was 129.6 nm (Fig. 1B). WB analysis showed that the exosome-specific markers CD63 and TSG101 were expressed in exosomes derived from U343-R and U343 cells (Fig. 1C). The above results proved that the extracted exosomes were standard exosomes. To identify differentially expressed circRNAs between TMZ-sensitive glioma cell-derived exosomes and TMZ-resistant glioma cell-derived exosomes, we performed microarray analysis of U343-R-exos and U343-exos. The volcano plot shows that thousands of circRNAs were differentially expressed in U343-R-exos compared to U343-exos (Fig. 1D). Hsa_circ_0050688 was found to be one of the most upregulated circRNAs (FC = 6.75, *P*-value < 0. 001) (Fig. 1E). Using the UCSC genome browser, we performed visual analysis of the structure of hsa_circ_0050688 and the genomic position and chromosomal localization of the host gene WDR62 (Fig. 1F). In addition, we graphically annotated the exons and introns of hsa_circ_0050688 with circPrimer software [25]. hsa_circ_0050688 was generated by back splicing of exon 8 to exon 15 of the WDR62 gene. The mature sequence length of this circRNA transcript was 1076 nt, and we termed hsa_circ_0050688 “circWDR62”. circWDR62 is located on chromosome 19, chr19:36562457-36580208 + (Fig. 1G). In addition, the stability of circWDR62 was assessed, and the results revealed that RNase R failed to digest circWDR62; however, the mRNA expression of WDR62 decreased dramatically after RNase R treatment (Fig. 1H).

As revealed by qRT–PCR, circWDR62 expression was significantly elevated in exosomes derived from serum samples from patients with recurrent glioma compared to those from patients with primary tumours, and circWDR62 expression in glioma patients was higher than that in normal controls (Fig. 1I). As shown in Fig. 1J, circWDR62 expression was higher in the tissues of patients with recurrent glioma than in those of patients with primary tumours. Additionally, the Kaplan–Meier survival curves demonstrated that the patients with recurrent glioma in the low circWDR62 expression group had a higher survival rate than those in the high circWDR62 expression group (Fig. 1K). We also investigated the relationship between circWDR62 expression and the clinicopathological characteristics of these patients, which are listed in Table 1. These results together suggest that high expression of exosomal circWDR62 is associated with malignant progression in glioma patients.

**Table 1:** The relationships between circWDR62 expression and the clinicopathological characteristics of glioma patients.

Characteristics	All patients	Relative circWDR62 level		*P*-value
		High	Low	
Age (years)				>0.05
≥50	33	18	15	
<50	27	14	13	
Gender				>0.05
Male	35	19	16	
Female	25	13	12	
Tumour size (diameter, cm)				<0.05
>5	36	24	12	
≤5	24	9	15	
WHO grade				<0.05
I–II	18	8	12	
III–IV	42	31	11	

### Knockdown of circWDR62 inhibits the TMZ resistance and malignant progression of glioma cells

To further explore the important biological functions of circWDR62, we evaluated the expression of circWDR62 in U343-R and U251-R cells as well as in their TMZ-sensitive counterparts. qRT–PCR data showed that circWDR62 expression was significantly increased in the two TMZ-resistant glioma cell lines (Additional file: Fig. S2A) and exosomes derived from these two cell lines compared to their TMZ-sensitive counterparts and exosomes derived from these cells (Additional file: Fig. S2B). Since circWDR62 expression is upregulated in TMZ-resistant cells, we used RNA interference to knock down the expression of circWDR62 to evaluate its pathological functions in glioma. qRT–PCR analysis demonstrated that circWDR62 expression was successfully inhibited (Additional file: Fig. S2C). Knockdown of circWDR62 reduced the IC50 of TMZ in U343-R and U251-R cells (Additional file: Fig. S2D). The CCK-8 assay demonstrated that downregulation of circWDR62 expression significantly suppressed the proliferation of U343-R and U251-R cells (Additional file: Fig. S2E). In addition, Our results showed that the invasion and migration of U343-R and U251-R cells were significantly decreased by circWDR62 expression downregulation (Additional file: Fig. S2F, G).

### circWDR62 promotes glioma TMZ resistance and malignant progression by acting as a sponge of miR-370-3p

**Fig. 2:**
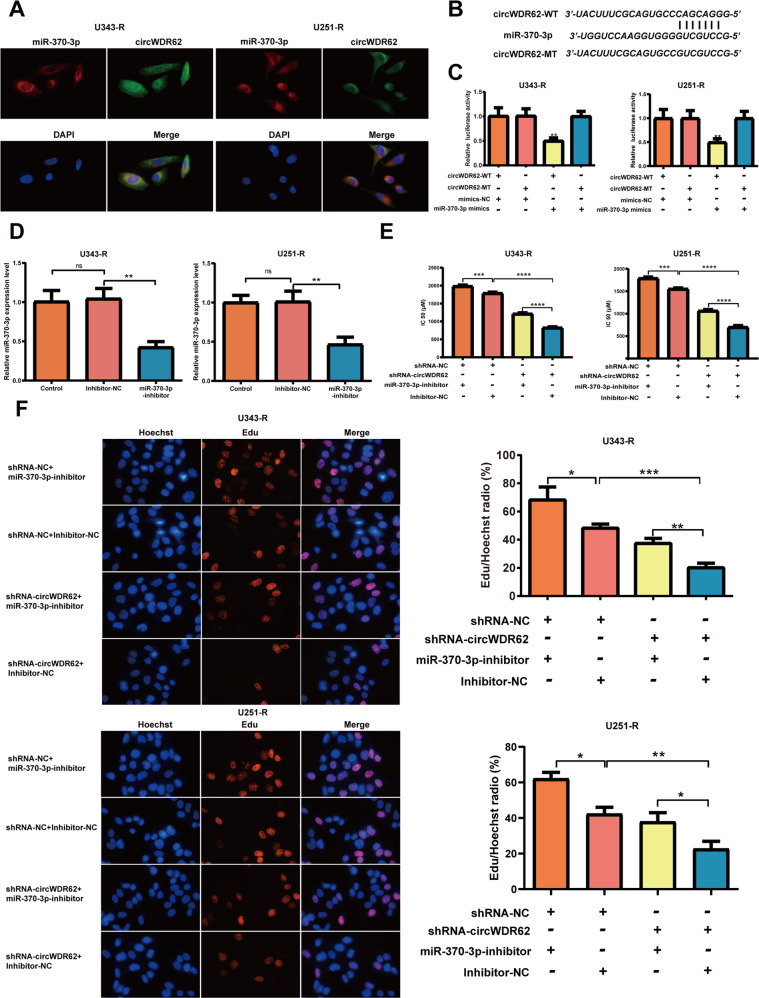
circWDR62 promotes TMZ resistance and cell proliferation by acting as a sponge of miR-370-3p. U343-R and U251-R cells were transfected with shRNA-NC or shRNA-circWDR62 and miR-370-3p inhibitor or inhibitor-NC. **A** Colocalization of circWDR62 and miR-370-3p was analysed in U343-R and U251-R cells by FISH. **B** The potential binding sites of miR-370-3p within the WT or mutant circWDR62 are shown. **C** 293 T cells were cotransfected with the WT circWDR62 reporter plasmid or the mutant circWDR62 reporter plasmid of and miR-370-3p mimic or mimic-NC, and luciferase activity was determined. **D** qRT–PCR analysis demonstrated that the transfection of miR-370-3p inhibitor was successful. **E** The IC50 of TMZ in each group was measured by the CCK-8 assay. **F** The EdU assay was performed to assess cell proliferation ability in each group.

To further elucidate the mechanism of action of circWDR62, we identified binding sites for many miRNAs in circWDR62 using the circBank database. We focused on 9 miRNAs that are closely related to glioma malignant progression and TMZ resistance and have strong binding ability as the key research objects for subsequent screening. We noted that among these miRNAs, miR-370-3p could bind with circWDR62, and the FISH results revealed that circWDR62 and miR-370-3p were colocalized in the U343-R and U251-R cell cytoplasm (Fig. 2A), indicating that circWDR62 may serve as a miRNA sponge in glioma. The potential binding site of miR-370-3p in circWDR62 was determined via bioinformatics analysis and is shown in the schematic in Fig. 2B. The results of the dual-luciferase reporter assay showed that overexpression of miR-370-3p by cotransfection of miR-370-3p mimics could decrease the luciferase activity of the WT circWDR62 reporter but not the mutant circWDR62 reporter, which indicated that miR-370-3p could directly bind to circWDR62 (Fig. 2C). Hence, our data illustrated that circWDR62 may function as a sponge of miR-370-3p in glioma.

**Fig. 3:**
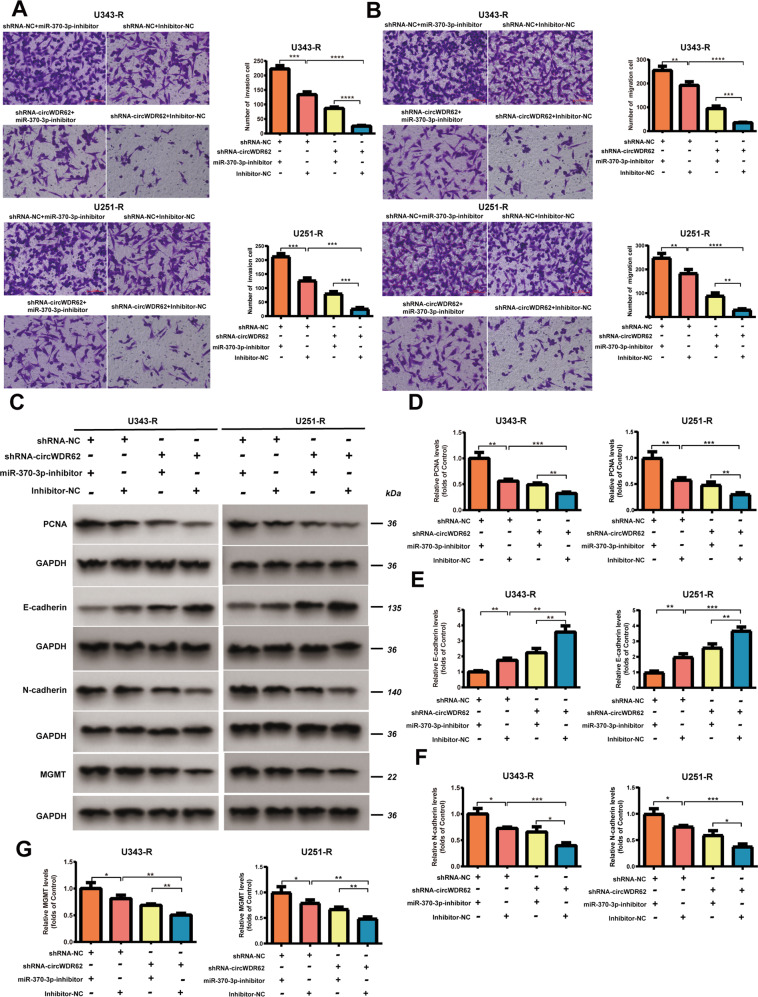
circWDR62 promotes cell invasion and migration by acting as a sponge of miR-370-3p. U343-R and U251-R cells were transfected with shRNA-NC or shRNA-circWDR62 and miR-370-3p inhibitor or inhibitor-NC. A-B Transwell assays were performed to assess the invasion (**A**) and migration (**B**) of U343-R and U251-R cells. **C**–**G** The protein expression levels of PCNA (**D**), E-cadherin (**E**), N-cadherin (**F**) and MGMT (**G**) were measured by WB analysis.

To explore the sponging mechanism of circWDR62, an shRNA targeting circWDR62 and an miR-370-3p inhibitor was designed. After transfection of shRNA-circWDR62, the expression of circWDR62 in U343-R and U251-R cells were signifcantly downregulated (Fig. 1L). However, suppression of circWDR62 did not change the WDR62 mRNA expression (Fig. 1M). qRT–PCR analysis demonstrated that the expression of miR-370-3p was downregulated in U343-R and U251-R cells after transfection of the miR-370-3p inhibitor (Fig. 2D), illustrating that miR-370-3p expression was successfully inhibited. There was a marked decrease in the IC50 of TMZ in U343-R and U251-R cells in the circWDR62 knockdown group compared with the negative control group, and inhibition of miR-370-3p significantly increased the IC50 of TMZ in U343-R and U251-R cells. However, the regulatory effects of circWDR62 knockdown were abrogated by cotransfection of the miR-370-3p inhibitor in U343-R and U251-R cells (Fig. 2E). The EdU assay revealed that cell proliferation was significantly decreased after knockdown of circWDR62. miR-370-3p suppression significantly increased the proliferation of U343-R and U251-R cells. In addition, miR-370-3p inhibition significantly reversed the circWDR62 suppression-induced inhibition of U343-R and U251-R cell proliferation (Fig. 2F). Transwell assays revealed that the migration and invasion of U343-R and U251-R cells were significantly decreased after knockdown of circWDR62 and that miR-370-3p suppression significantly increased the invasion and migration of U343-R and U251-R cells. miR-370-3p inhibition significantly reversed the circWDR62 suppression-induced inhibition of invasion and migration in U343-R and U251-R cells (Fig. 3A, B). WB analysis revealed that the protein levels of the target gene MGMT, proliferation-associated marker PCNA, and mesenchymal marker N-cadherin were significantly decreased in circWDR62-silenced cells, while the expression of the epithelial marker E-cadherin was increased. As expected, the miR-370-3p inhibitor significantly reversed the circWDR62 suppression-induced downregulation of MGMT, PCNA, and N-cadherin protein expression and upregulation of E-cadherin protein expression in U343-R and U251-R cells (Fig. 3C–G).

### MGMT is a direct target gene of the circWDR62/miR-370-3p axis in glioma

**Fig. 4:**
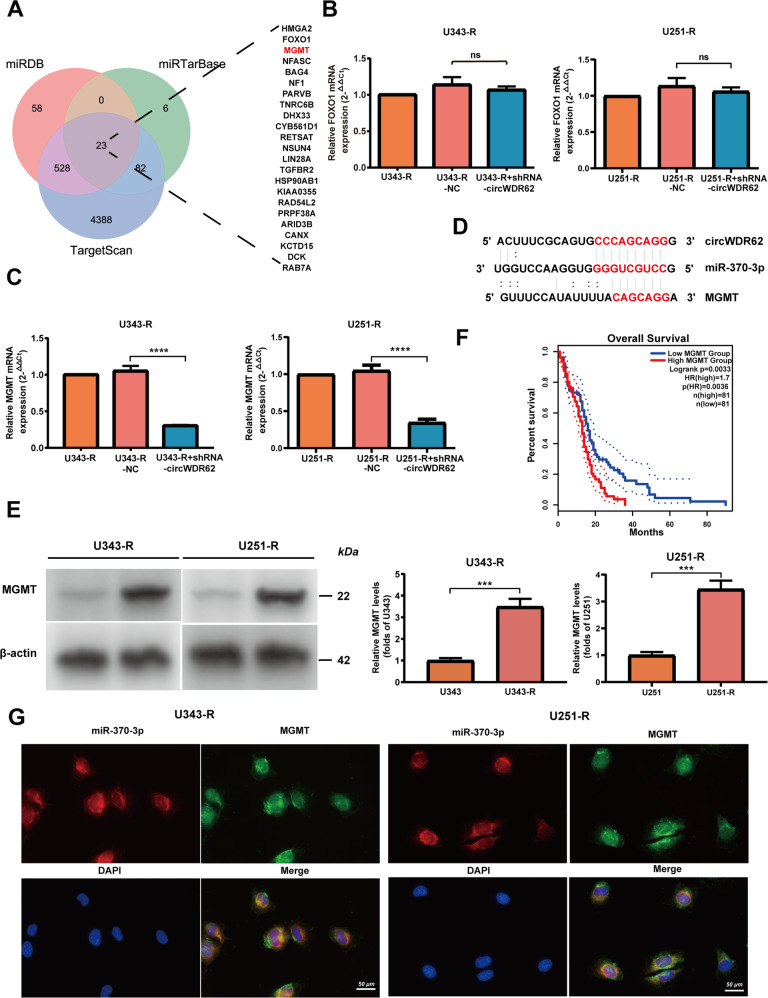
MGMT is a direct target gene of the circWDR62/miR-370-3p axis in glioma. **A** Venn diagram of 23 potential target mRNAs of miR-370-3p identified in three databases (miRDB, miRTarBase, and TargetScan). **B**, **C** Analysis of the expression of the potential targets FOXO1 (**B**) and MGMT (**C**) in the treated cells by qRT–PCR. **D** The binding sites of circWDR62, miR-370-3p and MGMT mRNA. **E** WB analysis was used to analyse the protein expression levels of MGMT in TMZ-resistant glioma cells and TMZ-sensitive glioma cells. **F** Prognostic analysis of MGMT expression by using the GEPIA2 database (http://gepia2.cancer-pku.cn/). **G** Colocalization of miR-370-3p and MGMT was analysed in U343-R and U251-R cells by FISH.

Three databases were used to predict the potential target mRNAs of miRNAs. The Venn diagram shows 23 mRNAs that are the most likely target genes of miR-370-3p (Fig. 4A). We further measured the expression of potential target genes after knockdown of circWDR62 and observed no significant change in FOXO1 expression (Fig. 4B) and decreased MGMT expression (Fig. 4C). Analysis of binding sites by circinteractome, miRBase and miRDB showed that circWDR62 could bind with miR-370-3p and that miR-370-3p could bind to the 3' UTR of MGMT mRNA (Fig. 4D). In addition, it has been reported that miR-370-3p can directly bind MGMT, which was validated by dual-luciferase reporter assays [26]. In addition, as shown in Fig. 4E, the protein expression of MGMT was upregulated in TMZ-resistant glioma cells compared with TMZ-sensitive glioma cells. Moreover, it was found that high MGMT expression was associated with a lower overall survival rate in glioma patients according to the GEPIA2 database (Fig. 4F). In addition, the FISH results revealed that miR-370-3p and MGMT were all colocalized in the U343-R and U251-R cell cytoplasm (Fig. 4G), which supporting our above results.

**Fig. 5:**
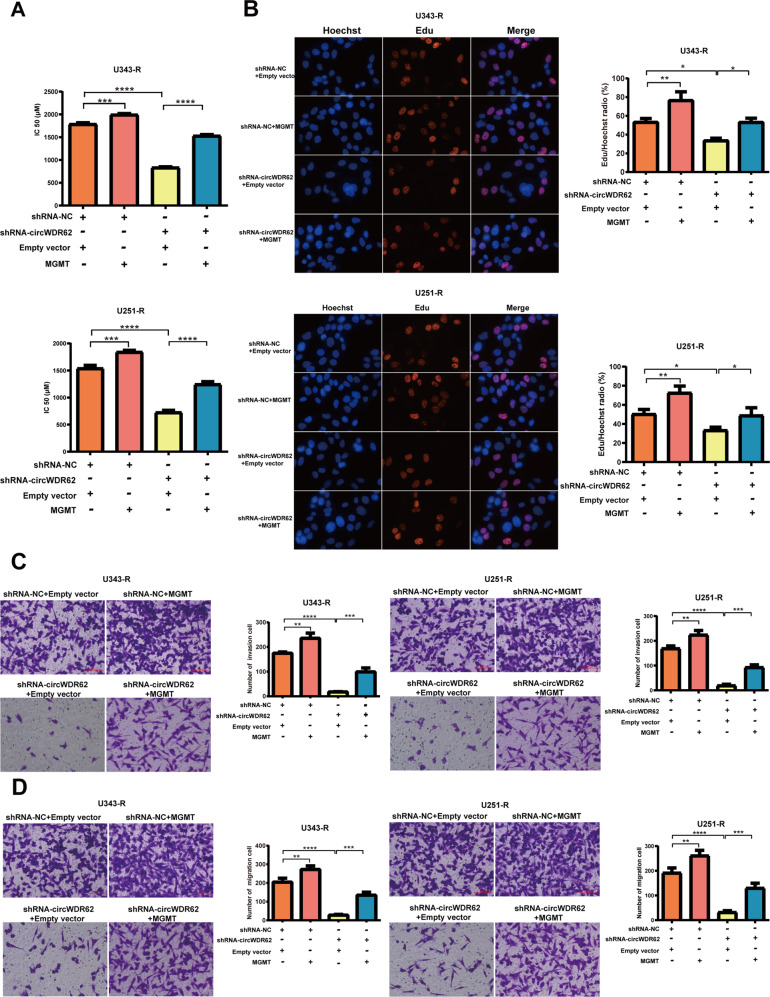
Overexpression of MGMT reversed circWDR62 knockdown-induced TMZ resistance and malignant progression of glioma cells. U343-R and U251-R cells were transfected with shRNA-NC or shRNA-circWDR62 and MGMT vector or empty vector. **A** The IC50 of TMZ was measured by the CCK-8 assay. **B** The EdU assay was performed to assess cell proliferation. **C**, **D** Transwell assays were performed to assess cell invasion (**C**) and migration (**D**).

Rescue assay revealed that MGMT overexpression significantly reversed the circWDR62 knockdown-induced suppression of TMZ resistance in U343-R and U251-R cells (Fig. 5A). In addition, MGMT overexpression significantly reversed the circWDR62 suppression-induced inhibition of U343-R and U251-R cell proliferation (Fig. 5B), invasion (Fig. 5C) and migration (Fig. 5D). Moreover, our data revealed that the protein expression of MGMT was upregulated after administration of the MGMT overexpression vector. Knockdown of circWDR62 obviously inhibited the protein expression of MGMT. MGMT overexpression significantly reversed the circWDR62 suppression-induced downregulation of MGMT, PCNA, and N-cadherin protein expression and upregulation of E-cadherin protein expression in U343-R and U251-R cells (Additional file: Fig. S3A–E).

In conclusion, these data support our hypothesis that MGMT is the downstream target gene of the circWDR62/miR-370-3p axis, providing further evidence for the role of circWDR62 as a ceRNA.

### circWDR62 enhances glioma TMZ resistance and tumour growth in vivo

**Fig. 6:**
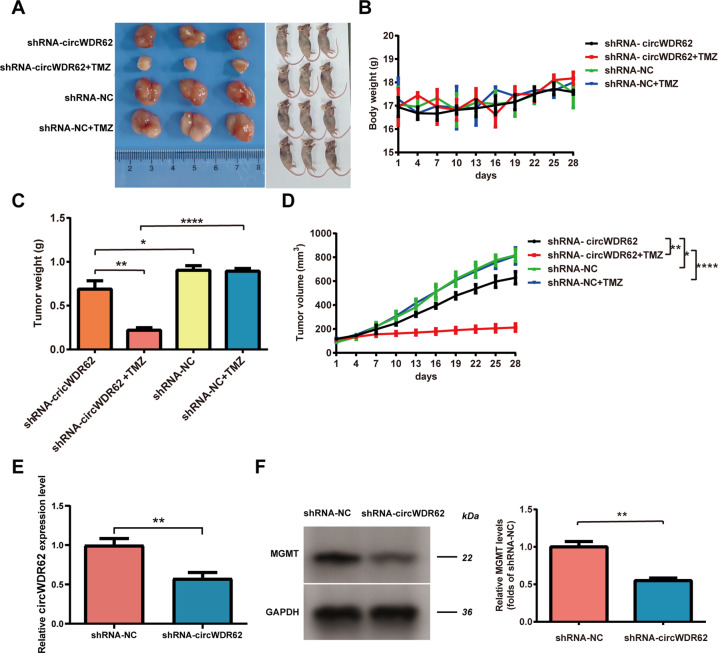
circWDR62 enhances glioma TMZ resistance and tumour growth in vivo. **A** Tumours from mice in different groups are shown. Tumour-bearing nude mice from different groups are shown. **B** Body weight changes in tumour-bearing mice. **C**, **D** The tumour weight (**C**) and tumour volume (**D**) of the mice were measured. **E** The expression of circWDR62 was measured in the tumour tissue from each group by qRT–PCR. **F** The MGMT protein expression level in tumour tissues was measured by WB analysis.

To investigate the relationship between circWDR62 and glioma TMZ resistance in vivo, 1 × 10^7^ U343-R cells transfected with shRNA-negative control (shRNA-NC) or lentivirus-shRNA-circWDR62 were injected subcutaneously into both armpits of nude mice to establish a xenograft tumour. TMZ was injected intraperitoneally into the nude mice once every 3 days. In the xenograft tumour model mice, the tumours derived from cells transfected with shRNA-circWDR62 were smaller than those derived from cells transfected with shRNA-NC. In addition, the proliferation rates of U343-R cells in vivo were reduced in response to TMZ treatment (Fig. 6A). There was no significant difference in the body weight of tumour-bearing mice between groups over time (Fig. 6B). However, knockdown of circWDR62 significantly suppressed the tumour weight and volume regardless of TMZ treatment (Fig. 6C, D). In addition, the expression of circWDR62 in tumour tissue was downregulated in the shRNA-circWDR62 group compared with the shRNA-NC group (Fig. 6E). Furthermore, WB analysis confirmed that MGMT protein expression was downregulated in tumour tissue in the shRNA-circWDR62 group compared to the shRNA-NC group (Fig. 6F).

### Exosome-mediated delivery of circWDR62 induces the TMZ resistance and malignant progression of glioma in vitro and in vivo

**Fig. 7:**
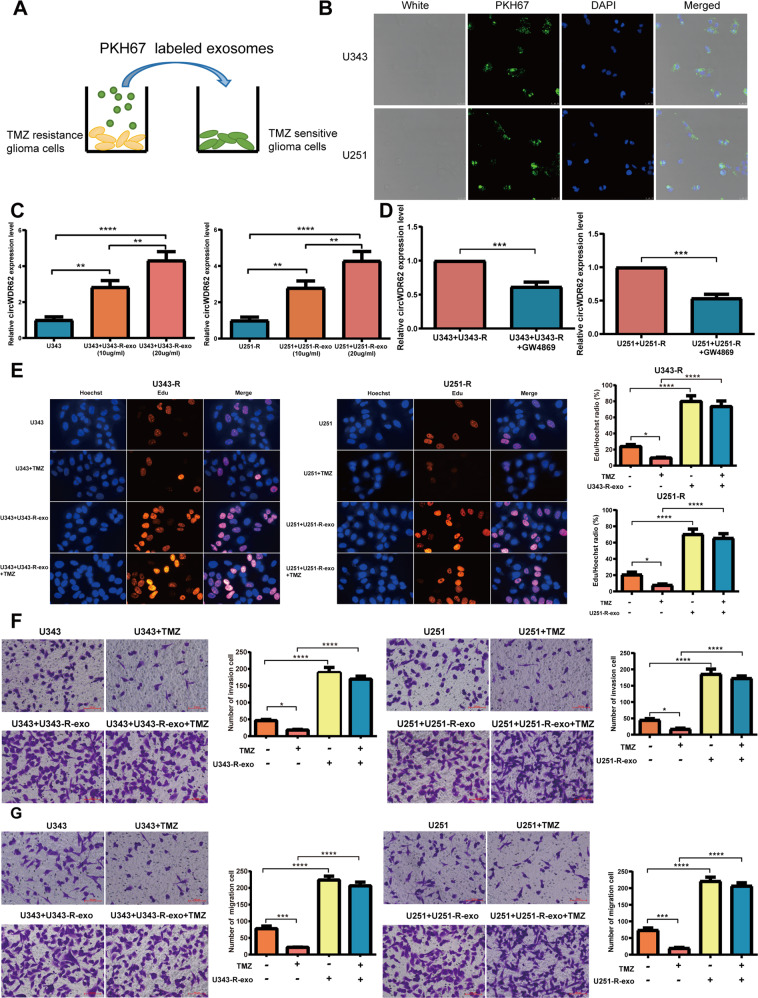
Exosome-mediated delivery of circWDR62 induces TMZ resistance and malignant progression of glioma cells in vitro. **A** Exosomes from TMZ-resistant glioma cells were labelled with PKH67 and then cocultured with TMZ-sensitive glioma cells. **B** PKH67-labelled TMZ-resistant glioma cell-derived exosomes were endocytosed by TMZ-sensitive glioma cells. **C** The expression levels of circWDR62 in TMZ-sensitive glioma cells were measured by qRT–PCR after treatment with TMZ-resistant glioma cell-derived exosomes. **D** The effects of the EV secretion inhibitor GW4689 on circWDR62 transport from U343-R and U251-R cells to U343 and U251 recipient cells. **E** The EdU assay was performed to assess the proliferation ability of TMZ-sensitive glioma cells after treatment with TMZ-resistant glioma cell-derived exosomes. **F**, **G** Transwell assays were performed to assess the cell invasion (**F**) and migration (**G**) of TMZ-sensitive glioma cells after treatment with TMZ-resistant glioma cell-derived exosomes.

**Fig. 8:**
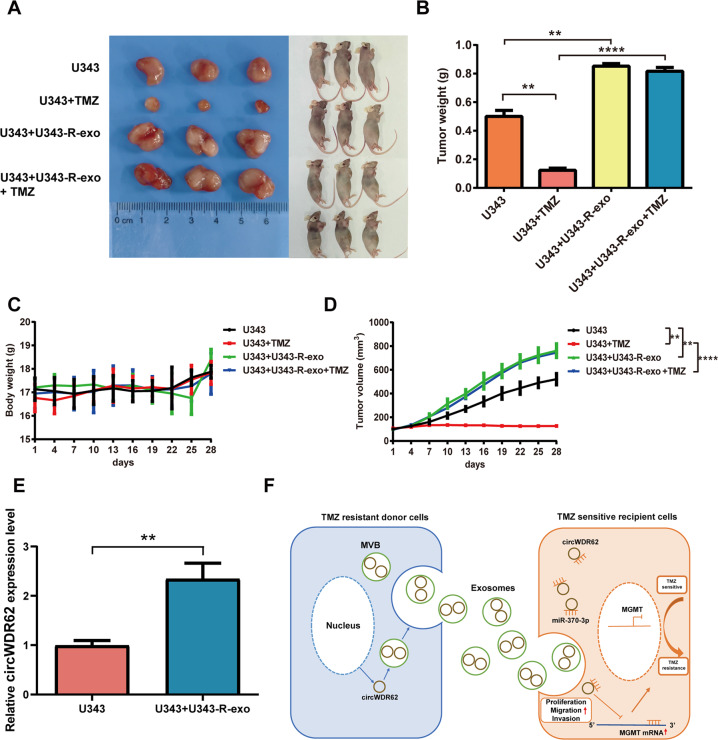
Exosome-mediated delivery of circWDR62 induces TMZ resistance and malignant progression of glioma in vivo. **A** Tumours of mice from different groups are shown. Tumour-bearing nude mice from different groups are shown. **B** The tumour weight of mice from each group was measured. **C** Body weight changes in tumour-bearing mice in each group. **D** The tumour volume of mice from each group was measured. **E** The expression of circWDR62 in each group was measured by qRT–PCR. **F** Schematic illustrating the biological function of the exosomal circWDR62/miR-370-3p/MGMT axis in glioma TMZ resistance and malignant progression.

Studies have confirmed that as important vehicles for intercellular communication, exosomes can transfer cargo from derived cells to target cells and thus play an important role in tumour generation, progression and chemotherapy resistance [22, 24, 27, 28]. In our study, TMZ-sensitive glioma cells were cultured with PKH67-labelled TMZ-resistant glioma cell-derived exosomes (Fig. 7A), and it was observed that TMZ-resistant glioma cell-derived exosomes were endocytosed by TMZ-sensitive glioma cells (Fig. 7B). As revealed by qRT–PCR, the expression levels of circWDR62 were significantly elevated by treatment with TMZ-resistant glioma cell-derived exosomes in TMZ-sensitive glioma cells and showed an upwards trend as the concentration of exosomes increased (Fig. 7C). However, after GW4869 was added, circWDR62 expression was significantly downregulated in the Transwell coculture system (Fig. 7D). These results indicate that circWDR62 can be transported from TMZ-resistant glioma cells to TMZ-sensitive glioma cells by incorporation into exosomes. In addition, the EdU assay and Transwell assays showed that the proliferation viability (Fig. 7E), invasion (Fig. 7F) and migration (Fig. 7G) of U343 and U251 cells were significantly increased by treatment with U343-R cell- and U251-R cell-derived exosomes, respectively. The results of the in vivo experiment showed that the tumour size (Fig. 8A), weight (Fig. 8B) and volume (Fig. 8D) of nude mice inoculated with U343 cells treated with U343-R cell-derived exosomes were significantly increased compared with those of nude mice inoculated with U343 cells. Furthermore, no significant difference was observed in the body weight of tumour-bearing mice between the groups (Fig. 8C). The expression of circWDR62 in tumour tissue from the U343 + U343-R-exo group was upregulated compared with that in the U343 group (Fig. 8E).

Hence, circWDR62 could be transported between TMZ-resistant and TMZ-sensitive glioma cells via exosomes. Exosomal circWDR62 from TMZ-resistant cells conferred TMZ resistance in recipient sensitive cells while also enhancing cell proliferation, migration and invasion of recipient sensitive cells. In summary, we illustrated that exosomal circWDR62 contributed to the TMZ resistance and malignant progression of glioma by sponging miR-370-3p via modulation of MGMT expression (Fig. 8F).

## Discussion

Although surgical techniques and radiochemotherapy regimens have been improving in recent decades, the survival rate of glioma has not increased as expected [29–32]. Chemoresistance is a major cause of chemotherapy failure in glioma patients. Understanding the mechanisms underlying resistance to TMZ in glioma and exploring new strategies to reverse TMZ resistance in glioma may contribute to the development of better treatment strategies to improve the prognosis of glioma.

Accumulating evidence has revealed that exosomes are involved in the chemotherapy resistance of tumour cells [33–35]. For example, a few studies have reported that exosomes derived from tumour cells regulate glioma malignant progression and TMZ resistance by delivering lncRNAs or miRNAs to recipient cells [36, 37]. circRNAs were been found to be delivered by exosomes and play an important regulatory role in glioma tumour proliferation, invasion and metastasis [38]. However, to date, only a few studies have proven that circRNAs are associated with chemoresistance in glioma, and studies on the role of exosome-mediated delivery of circRNAs in regulating resistance to chemotherapy are still scarce.

In the present study, microarray analysis was first performed on TMZ-sensitive and TMZ-resistant glioma exosomes to identify differentially expressed circRNAs. We identified a novel circRNA, termed circWDR62, and confirmed that the expression of circWDR62 was significantly upregulated in exosomes derived from TMZ-resistant glioma cells and serum samples from patients with recurrent glioma. Previous evidence indicates that the expression of the host gene WDR62 is significantly upregulated in most tumours and correlates with poor prognosis in multiple cancers [39, 40]. Subsequently, an RNase R treatment assay was carried out to confirm the stability of circWDR62. Because circWDR62 has not been reported to be associated with chemoresistance, we are the first to elucidate the role of circWDR62 in TMZ resistance in glioma.

Our results confirmed that circWDR62 is a chemoresistance-associated circRNA and can promote the TMZ resistance and malignant progression of glioma. In addition, the subcellular localization of circRNAs is closely related to their biological function. Herein, we found that circWDR62 is mainly a cytoplasmic circRNA in glioma cells. Accumulating evidence indicates that sponging of miRNAs by cytoplasmic circRNAs is the most common mechanism [41, 42]. For example, circCRIM1 acts as a ceRNA for the miR-422a-FOXQ1 axis, ultimately leading to nasopharyngeal carcinoma metastasis, EMT, and docetaxel chemotherapy resistance [43]. Thus, we further explored whether circWDR62 exerts its regulatory effects by acting as a miRNA sponge. The miRNAs that may interact with circWDR62 were identified using an online database. In 9 predicted candidate miRNAs (hsa-miR-489-3p, hsa-miR-320e, hsa-miR-370-3p, hsa-miR-485-3p, hsa-miR-490-5p, hsa-miR-503-3p, hsa-miR-508-5p, hsa-miR-93-5p and hsa-miR-98-5p), none of the other 8 miRNAs have been reported to be associated with TMZ resistance. Of these predicted candidate miRNAs, miR-370-3p was of particular interest in our study, as it is considered to be closely related to glioma malignant progression and TMZ resistance [26, 44]. Lulli et al. reported that upregulation of miR-370-3p expression can significantly decrease the proliferation, migration, and clonogenic abilities of GBM stem-like cells in vitro and tumour growth in vivo [45]. We are the first to highlight that circWDR62 may exert its regulatory effect by acting as a miR-370-3p sponge. circRNA could serve as a ceRNA to regulate the expression of target genes. Dual-luciferase reporter assays have revealed that miR-370-3p can directly bind MGMT [26]. MGMT is a critical DNA repair gene, and evidence indicates that MGMT expression correlates with chemoresistance and poor prognosis in multiple cancers, including glioma [46, 47]. In our study, bioinformatics analysis, rescue assays and in vivo experiments all confirmed that MGMT is a direct target gene of the circWDR62/miR-370-3p axis in glioma. We are the first to highlight that circWDR62 can directly target miR-370-3p and upregulate MGMT expression to promote TMZ resistance, proliferation, migration and invasion.

Exosomes can deliver circRNAs to promote or inhibit cancer development [48]. A few studies have confirmed that exosome-derived circRNAs promote chemoresistance in tumour cells [33, 49]. Our results demonstrate for the first time that circWDR62 can be transported via exosomes and that exosomal circWDR62 from TMZ-resistant cells confers TMZ resistance in recipient sensitive cells while also enhancing the proliferation, migration and invasion of these cells. These findings suggest that exosomal circWDR62 plays an important role in the transmission of chemoresistance in glioma. Previously, Ding et al. [50] found that the expression of circNFIX is increased in serum exosomes from TMZ-resistant glioma patients and that exosomal circNFIX can promote cell migration and invasion and inhibit apoptosis following TMZ exposure by competitively binding to miR-132, thus conferring sensitive cells with TMZ resistance. Recently, Ding et al. reported that exosomal circ_0072083 can enhance TMZ resistance by targeting NANOG [51]. Therefore, more chemoresistance-associated circRNAs need to be identified in the future.

Nonetheless, there are limitations to this study. First, circWDR62 was demonstrated to function as a miRNA sponge, but whether circWDR62 can regulate chemoresistance by binding to RBPs or through other epigenetic mechanisms was not explored [52]. Second, we focused on the roles of circWDR62 in TMZ resistance and malignant progression. More detailed studies are necessary to explore the role of circWDR62 in other biological behaviours of glioma, including immune escape and tumour energy metabolism [53]. In addition, the upstream regulatory mechanism of circWDR62 in TMZ-resistant glioma cells and tissues also worthy of further exploration.

## Conclusions

In conclusion, our study demonstrated that novel chemoresistance-associated circWDR62 can be transported via exosomes and that exosomal circWDR62 might function as a miR-370-3p sponge to regulate MGMT expression, promoting the TMZ resistance and malignant development of glioma in vitro and in vivo. Our findings highlight a novel direction underlying the development of TMZ resistance in glioma and provide a new potential target for the treatment of TMZ-resistant glioma.

## Materials and methods

### Clinical glioma patient samples

A total of 60 glioma patients, including 32 patients with primary tumours and 28 patients with recurrent tumours after treatment with TMZ-based chemotherapy, were enroled in this study between 2017 and 2020. Tumour tissues and peripheral blood samples (10 ml) were collected. The tissues were immersed in liquid nitrogen immediately after removal from patients, and serum specimens were obtained by centrifugation and then kept in a refrigerator at −80 °C. Fifteen healthy volunteers were included as normal controls. The mean exosomal circWDR62 level in the serum samples was chosen as the cut-off point, and the 28 patients with recurrent tumours were separated into the high circWDR62 expression group (*n* = 15) and the low circWDR62 expression group (*n* = 13). The research was carried out in accordance with principles of the World Medical Association Declaration of Helsinki. Clinicopathological classification and staging were performed according to the American Joint Committee on Cancer Classification Criteria. Written informed consent was obtained from all patients or their legal guardians, and our experiments were approved by the Medical Ethics Committee of the Affiliated Hospital of Hebei University.

### Cell culture and establishment of TMZ-resistant cell lines

Stable TMZ-resistant U343 and U251 glioma cells were generated by gradient induction and named U343-R cells and U251-R cells, respectively. Glioma cells were all recently authenticated by STR profiling. TMZ (T2577, Sigma–Aldrich, St Louis, USA) was dissolved in DMSO and stored at −20 °C. The concentrations of TMZ that U343 and U251 cells were exposed to were gradually increased, with the cells being exposed to each dose for 20 days and the whole induction process lasting for <6 months. Glioma cells were maintained in high-glucose Dulbecco’s modified Eagle medium (DMEM, Gibco, Grand Island, USA) supplemented with antibiotics (100 units/mL penicillin and 100 mg/mL streptomycin) and 10% foetal bovine serum (FBS, HyClone, Logan, UT, USA). All the cells were maintained at 37 °C in a humidified incubator containing 5% CO_2_.

### Exosome isolation and identification

Exosomes were isolated from cell culture medium containing 10% exosome-free FBS (SBI: EXO-FBS-50A-1) by ultracentrifugation (Hitachi CP100MX ultracentrifuge). Cell culture medium was collected and centrifuged at 300 × *g* for 10 min, 2000 × *g* for 10 min and 10,000 × *g* for 30 min to remove residual live cells, dead cells and cell debris, respectively. The supernatant was then collected and centrifuged at 110,000 × *g* for 90 min at 4 °C to precipitate the exosomes. Exosome precipitates were washed with phosphate-buffered saline (PBS) for purification and then resuspended in PBS for further analysis.

Exosomes were visualized using transmission electron microscopy (TEM; Hitachi, Japan). The concentration and size distribution of the isolated exosomes were determined using nanoparticle tracking analysis according to the manufacturer’s guidance. NTA was performed using a ZetaView instrument (Particle Metrix, Meerbusch, Germany) and corresponding software (ZetaView 8.04.02). Western blot (WB) analysis was performed to measure the levels of the exosome protein markers CD63 (ab216130, 1:2000 dilution) (Abcam, Cambridge, UK) and TSG101 (ab125011, 1:10,000 dilution) (Abcam, Cambridge, UK).

### Microarray data analysis

Total RNA was extracted and purified using a Qiagen Serum/Plasma Kit (Cat# 217184, Qiagen) following the manufacturer’s instructions, and the RNA integrity number (RIN) was determined by using an Agilent 2100 Bioanalyzer (Agilent Technologies, Santa Clara, CA, US). The data were extracted with Feature Extraction software 10.7 (Agilent Technologies, Santa Clara, CA, US). The raw data were normalized by the quantile algorithm and limma packages in R. circRNAs that exhibited a fold change in expression ≥ 2.0 and a *P*-value < 0.05 were recognized as significantly differentially expressed. The microarray analysis was conducted by Shanghai Biotechnology Corporation (Shanghai, China).

### RNA extraction and quantitative real-time polymerase chain reaction (qRT–PCR)

Total RNA was extracted from exosomes, cells and tissues using TRIzol reagent (Invitrogen, Carlsbad, CA, USA) based on the manufacturers’ instructions. For the measurement of circWDR62 levels, cDNA was synthesized from total RNA using PrimeScript RT Master Mix (Takara Bio., Japan). qRT–PCR was performed with TB Green Premix Ex Taq™ II (Tli RNaseH Plus) (Takara Bio., Japan). GAPDH was used as the internal reference. The relative expression levels of circWDR62, miR-370-3p and MGMT were calculated by the 2 − ΔΔCt method. The primer sequences (5′-3′) used for qRT–PCR are listed in Supplementary Table S1.

### RNase R treatment assay

Total RNA was incubated at 37 °C with 3 U/ug of RNase R (Epicenter Technologies, Madison, WI, USA) for 30 min in accordance with the manufacturer’s protocol. qRT–PCR was performed to assess the mRNA expression levels of circWDR62 and WDR62.

### Cell transfection

A small interfering RNA (siRNA) targeting circWDR62 (siRNA-circWDR62) and a matched negative control siRNA (siRNA-NC) were synthesized by RiboBio (Guangzhou, China). An miR-370-3p inhibitor and matched negative control (inhibitor-NC) were synthesized by Thermo Fisher Scientific. Lentivirus-packaged shRNA-circWDR62 and its negative control (shRNA-NC) were provided by GeneChem Company (Shanghai, China). MGMT overexpression vectors and an empty vector were synthesized by Addgene (Cambridge, USA). Cells were transfected by using Lipofectamine 3000 (Thermo Fisher Scientific, USA). The oligo sequences used for transfection in this research are listed in Supplementary Table S2.

### Assessment of cell sensitivity to TMZ

To calculate the half maximal inhibitory concentration (IC50) of cells, the CCK-8 assay (Beyotime Biotechnology, China) was used to measure cell viability. DMEM containing different concentrations of TMZ was added to cells according to the experimental group, and the cells were incubated for 48 h. The concentrations of TMZ were 0 μM, 25 μM, 50 μM, 100 μM, 200 μM, 400 μM, 800 μM, and 1600 μM. Three replicates were treated with medium containing each concentration of TMZ. Before the chemotherapy sensitivity of the cells was assessed, 10 μl CCK-8 reagent was added to each well before the end of drug incubation, and then the cells were cultured for another 4 h. Subsequently, absorption at 450 nm was measured using a Berthold LB941 microplate multifunctional enzyme reader. The survival rate (%) was calculated according to the following formula: survival rate (%) = mean optical density (OD) of the experimental group/mean OD of the control group × 100. The cell inhibition rate (%) was calculated as 100-the survival rate (%). The RI was calculated as the IC50 of resistant cells/the IC50 of sensitive cells.

### Cell counting kit-8 (CCK-8) assay

Cell proliferation was assessed by Cell Counting Kit-8 (CCK-8) assay (Beyotime Biotechnology, China). Cells were seeded in 96-well plates and incubated at 37 °C for 24 h before transfection. Ten microlitres of CCK-8 reagent was added to each well 48 h after transfection. After 2 h of incubation at 37 °C, the absorbance at 450 nM was measured using a microplate reader (TriStar LB941, Berthold Technologies).

### 5-Ethynyl-2′-deoxyuridine (EdU) assay

The EdU assay was performed using a Cell-Light EdU DNA Cell Proliferation Kit (Ribo Life Science, Suzhou, China) according to the manufacturer’s protocol. A total of 4 × 10^4^ cells were seeded in 96-well plates and cultured for 48 h. After incubation with 100 μL of diluted EdU-containing medium (1000:1) for 2 h, the cells were fixed in 4% paraformaldehyde and stained with Apollo Dye Solution. Hoechst 33342 was used to stain nucleic acids within the cells. EdU-positive cells were visualized and captured using a Zeiss LSM confocal microscope (Carl Zeiss Microscopy GmbH, Germany).

### Transwell assay

Transwell invasion and migration assays were performed in 24-well plates (Corning, MA, USA) using Transwell chambers (Millipore, Billerica, MA, USA). After 48 h of transfection, cells (2.5 × 10^5^) were plated in the upper chambers coated with (invasion assay) or without (migration assay) 40 μl of Matrigel (BD Biosciences, NJ, USA) in serum-free medium. Then, 600 μL high-glucose DMEM containing 10% FBS was loaded in the lower chambers. After incubation for 24 h at 37 °C, the cells were fixed with methanol, stained with 0.1% crystal violet solution for 15 min, and counted under a microscope. The numbers of cells counted in 5 random fields under a microscope were averaged.

### Fluorescence in situ hybridization (FISH) assay

A FITC-labelled probe for circWDR62 or MGMT and Texas Red-labelled probe for miR-370-3p were designed and synthesized by RiboBio, and the signals were detected with a FISH Kit (RiboBio, Guangzhou, China) according to the manufacturer’s instructions. When they reached 60–70% confluence, cells attached to the slides were immobilized with 4% paraformaldehyde and washed with PBS. After permeabilization (1× PBS/0.5% Triton X-100), hybridization buffer with specific probes was dripped onto the cell slide, and hybridized was performed overnight at 37 °C. The hybridization buffer was then gradually washed off with 4× SSC, 2× SSC and 1× SSC at 42 °C. Nuclei were counterstained with 4,6-diamidino-2-phenylindole (DAPI) at room temperature. Images were acquired using a fluorescence microscope.

### Luciferase reporter assay

For the luciferase reporter assay, pmirGLO dual-luciferase vectors (Promega) were used to construct dual-luciferase reporter plasmids. Wild-type (WT) and mutant (MT) circWDR62 3' UTRs were amplified and inserted into the luciferase reporter vector pmirGLO (Promega). HEK293T cells were cotransfected with luciferase plasmids and miR-370-3p mimics. After 48 h, the luciferase activity was measured with the Dual-Luciferase Reporter Gene Assay Kit (Beyotime, Shanghai, China) according to the manufacturer’s instructions, and firefly luciferase activity was normalized to Renilla luciferase activity.

### Western blot (WB) analysis

Protein was extracted from cells and mouse tissues using RIPA lysis buffer (Beyotime, China), and the protein concentration was quantified using a BCA protein assay kit (Thermo Scientific, USA). The proteins were separated by SDS–PAGE and transferred onto 0.45 μm PVDF membranes (Millipore, Schwalbach, Germany). The membranes were immersed in Tris-buffered saline-Tween 20 (TBST) blocking solution containing 5% nonfat milk powder and incubated with a primary antibody at 4 °C overnight, followed by a secondary antibody. Finally, the bands were visualized by using a Tanon 6600 Luminescence imaging workstation (Tanon, China) and analysed by Image-Pro Plus 6.0 software. The antibodies used included primary antibodies against PCNA (13110, 1:1000 dilution), E-cadherin (14472, 1:1000 dilution), N-cadherin (13116, 1:1000 dilution) (Cell Signalling Technology, Boston, USA), β‐actin (ab8226, 1:1000 dilution) and MGMT (ab108630, 1:1000 dilution) (Abcam, Cambridge, UK).

### Exosome-tracing and exosome secretion blocking experiments

For exosome-tracing experiments, isolated exosomes were labelled with PKH67 fluorescent dye using a PKH67 fluorescent cell linker kit (Sigma–Aldrich) following the manufacturer’s instructions. U343 and U251 cells were incubated with PKH67-labelled U343-R and U251-R cell-derived exosomes for 12 h, fixed with 4% paraformaldehyde, and then washed 3 times with PBS containing 0.1% Triton X-100. The cells were stained with DAPI and visualized with a laser confocal microscope (Carl Zeiss Microscopy GmbH, Germany). Exosome secretion was blocked using GW4869 (Med Chem Express, MCE), a specific chemical inhibitor of nSMase2.

### Bioinformatics analysis

Annotation of circWDR62 was performed based on data in circBase. The miRNAs that bind circWDR62 were predicted with the circbank database. The target genes of miR-370-3p were predicted with the miRDB, miRTarBase and TargetScan databases, and the intersecting genes were recorded. circRNA, miRNA and mRNA binding sites were analysed with circinteractome, miRBase and miRDB.

### Animal experiment

All animal experiments were performed according to the guidelines for animal experiments of the Affiliated Hospital of Hebei University and approved by the Animal Ethical Committee of the Affiliated Hospital of Hebei University. We chose 5- to 6-week-old male BALB/c nude mice for tumour xenograft experiments to study the effect of circWDR62 on TMZ resistance and tumour growth. The BALB/c nude mice used in this research were provided by Changzhou Cavins Experimental Animals Co., Ltd. In the in vivo tumour growth assay, U343-R cells stably transfected with lentivirus containing shRNA-circWDR62 or shRNA-NC were subcutaneously injected into the right axilla of nude mice (5 × 10^7^ cells/mL, 0.2 mL per mouse). Seven days after tumour formation, we intraperitoneally injected the mice with TMZ (20 mg/kg) in DMSO or DMSO alone every 3 days for a total of seven times. A total of 60 μg U343-R exosomes per mouse were injected intratumourally at each administration. The injections were repeated every 3 days, and the body weight of the mice was measured every 3 days until the termination of the experiment. The tumour volume was calculated as 1/2 × (length × width^2^) by using a calliper. Finally, the mice were sacrificed, and subcutaneous tumour tissues were collected to determine tumour weight and for WB analysis. (*n* = 6 for mice upon each treatment).

### Statistical analysis

Graph Pad Prism 8 and Statistical Product and Service Solutions (SPSS) software version 20.0 were used for statistical analysis. All experiments were performed in triplicate, and the results are expressed as the mean ± standard deviation (SD). Student’s *t*-test was used to analyse the significance of differences between two independent groups. One-way ANOVA was applied to analyse the differences among three or more groups. The relationships between the circWDR62 expression level and clinicopathological features of the glioma patients were analysed by the *χ*2-test. Survival analysis was performed with the Kaplan–Meier method. The differences in survival between the two groups were assessed using the log-rank test. *P*-values < 0.05 were considered statistically significant.

## Supplementary information

FigureS1

FigureS2

FigureS3

TableS1

TableS2

Reproducibility checklist

Original Data File

AJE Editing Certificate

## Data Availability

The datasets generated or analysed in the current study are available from the corresponding author upon reasonable request.
