# Supplementary figures and images for: Exosomal circWDR62 promotes temozolomide resistance and malignant progression through regulation of the miR-370-3p/MGMT axis in glioma
Source: Cell Death Dis. 2022 Jul 11;13(7):596. doi: 10.1038/s41419-022-05056-5 (PMC9273787; doi:10.1038/s41419-022-05056-5)

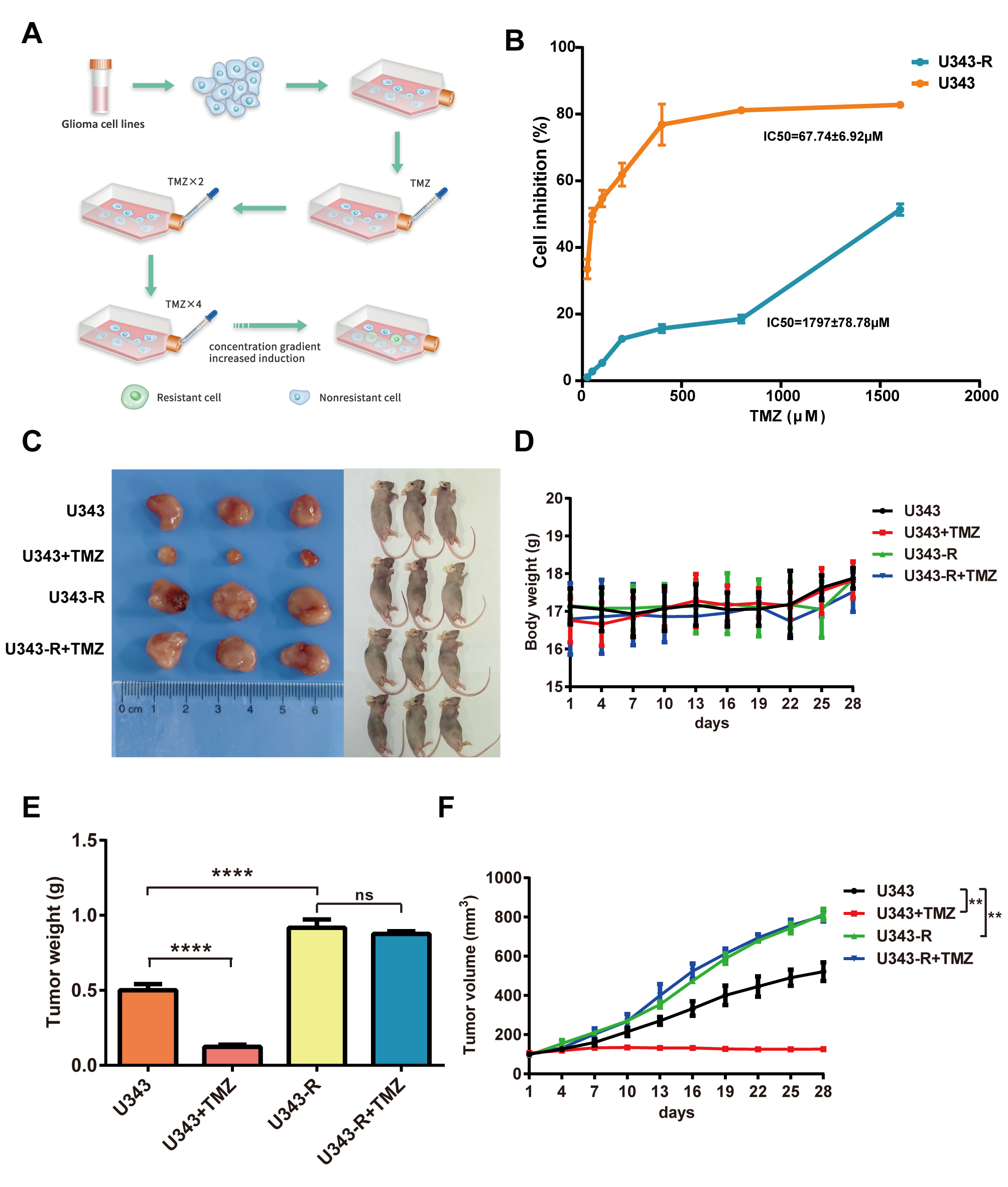

Supplement: Supplementary file 1 — FigureS1 [file 41419_2022_5056_MOESM1_ESM.tif]

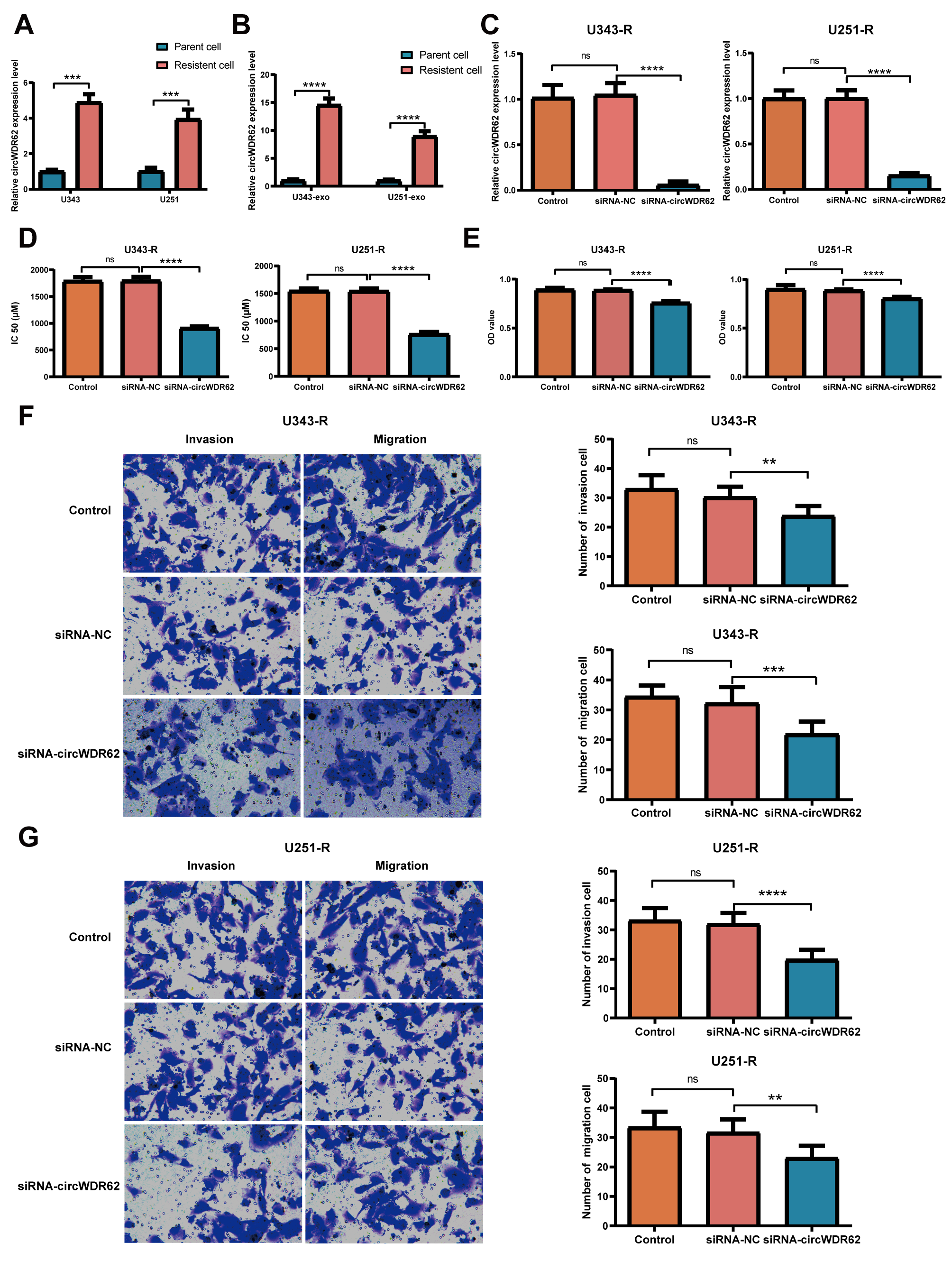

Supplement: Supplementary file 2 — FigureS2 [file 41419_2022_5056_MOESM2_ESM.tif]

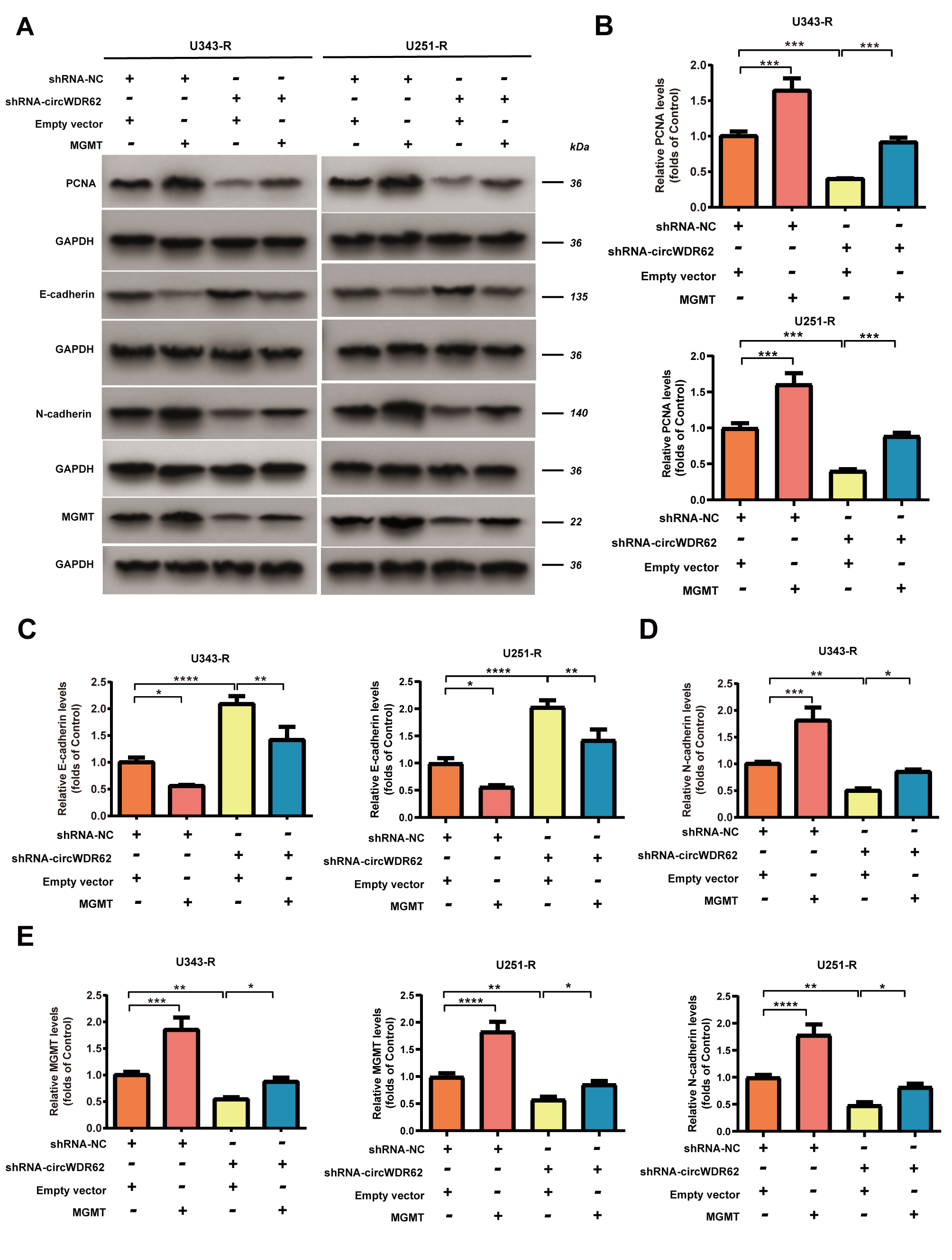

Supplement: Supplementary file 3 — FigureS3 [file 41419_2022_5056_MOESM3_ESM.tif]
