# Supplementary material for: Exosomal circWDR62 promotes temozolomide resistance and malignant progression through regulation of the miR-370-3p/MGMT axis in glioma
Source: Cell Death Dis. 2022 Jul 11;13(7):596. doi: 10.1038/s41419-022-05056-5 (PMC9273787; doi:10.1038/s41419-022-05056-5)
Supplement: Supplementary file 4 — TableS1 [file 41419_2022_5056_MOESM4_ESM.docx]

**Supplementary Table 1. The primer sequences (5′-3′) used for qRT–PCR in this research.**

| Name | Sequences (5’-3’) | |
| --- | --- | --- |
|  | Forward | Reverse |
| circWDR62 | CGCAATGTGAGGTCTCCC | GCCACAGAAGATGAGCTCCT |
| miR-370-3p | TGTAACCAGAGAGCGGGATGT | TTTTGGCATACTAAGGCCGAA |
| MGMT | ACCGTTTGCGACTTGGTACT | TGCTCACAACCAGACAGCTC |
| GAPDH | GCACCGTCAAGGCTGAGAAC | TGGTGAAGACGCCAGTGGA |
