# Supplementary material for: Exosomal circWDR62 promotes temozolomide resistance and malignant progression through regulation of the miR-370-3p/MGMT axis in glioma
Source: Cell Death Dis. 2022 Jul 11;13(7):596. doi: 10.1038/s41419-022-05056-5 (PMC9273787; doi:10.1038/s41419-022-05056-5)
Supplement: Supplementary file 5 — TableS2 [file 41419_2022_5056_MOESM5_ESM.docx]

**Supplementary Table 2. The oligo sequences used for transfection in this research.**

| Name | Sequence (5’-3’) |
| --- | --- |
| siRNA-circWDR62 | TGTGAGGTCTCCCTGTCTT |
| siRNA-NC | synthesized by RiboBio (Guangzhou, China) |
| sh-circWDR62 | GTGAGGTCTCCCTGTCTTCCT |
| shRNA-NC | CAACAAGATGAAGAGCACCAA |
| miR-370-3p miRNA mimic | GCCUGCUGGGGUGGAACCUGGU |
| mimic NC | CAGUACUUUUGUGUAGUACA |
| miR-370-3p miRNA inhibitor | GCCUGCUGGGGUGGAACCUGGU |
| inhibitor NC | CAGUACUUUUGUGUAGUACAA |
| MGMT-F | CTAGCTAGC ATGGACAAGGATTGTG |
| MGMT-R | CCCAAGCTT TCAGTTTCGGCCAGCAG |
