# Supplementary material for: Exosomal circWDR62 promotes temozolomide resistance and malignant progression through regulation of the miR-370-3p/MGMT axis in glioma
Source: Cell Death Dis. 2022 Jul 11;13(7):596. doi: 10.1038/s41419-022-05056-5 (PMC9273787; doi:10.1038/s41419-022-05056-5)

CD63 (Figure1)

CD63 26kDa -

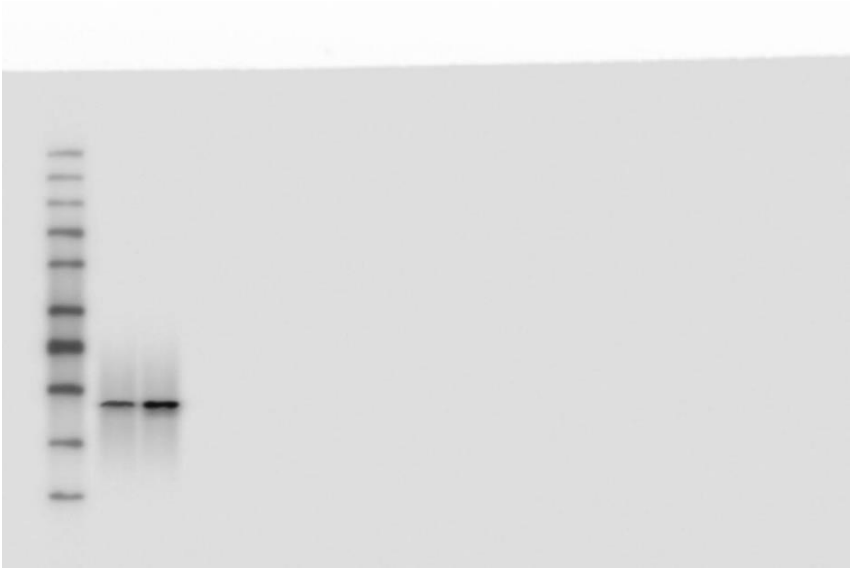

TSG101 (Figure1)

TSG101 44kDa -

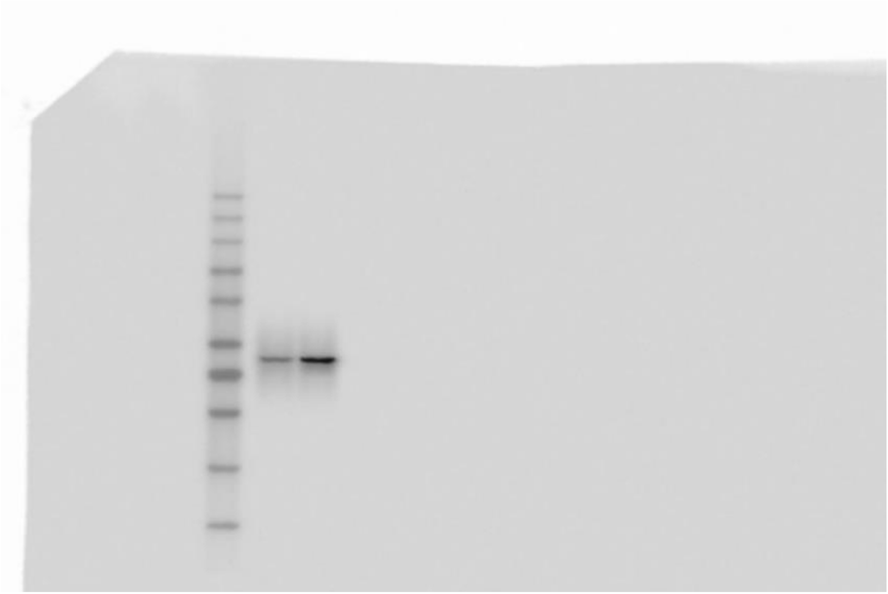

PCNA—U343-R (Figure3)

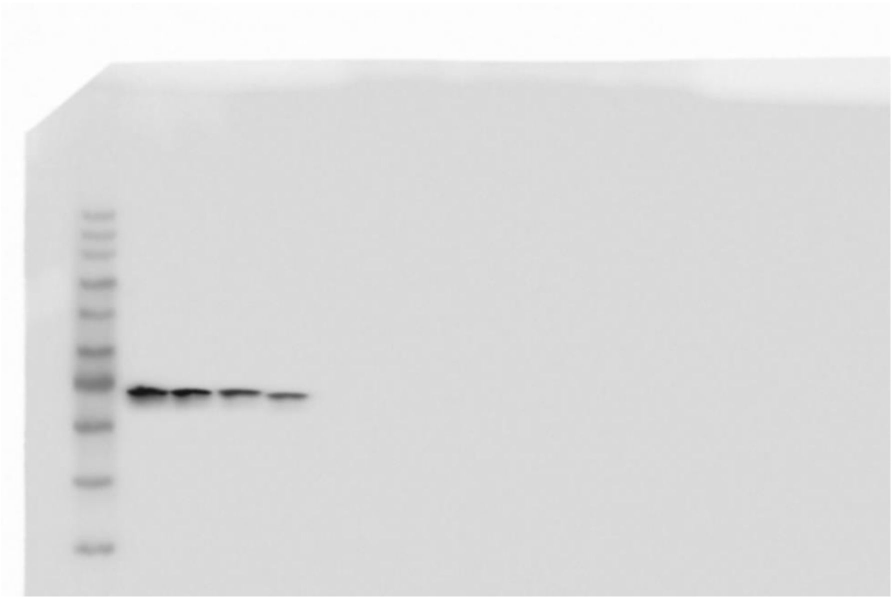

PCNA—U251-R (Figure3)

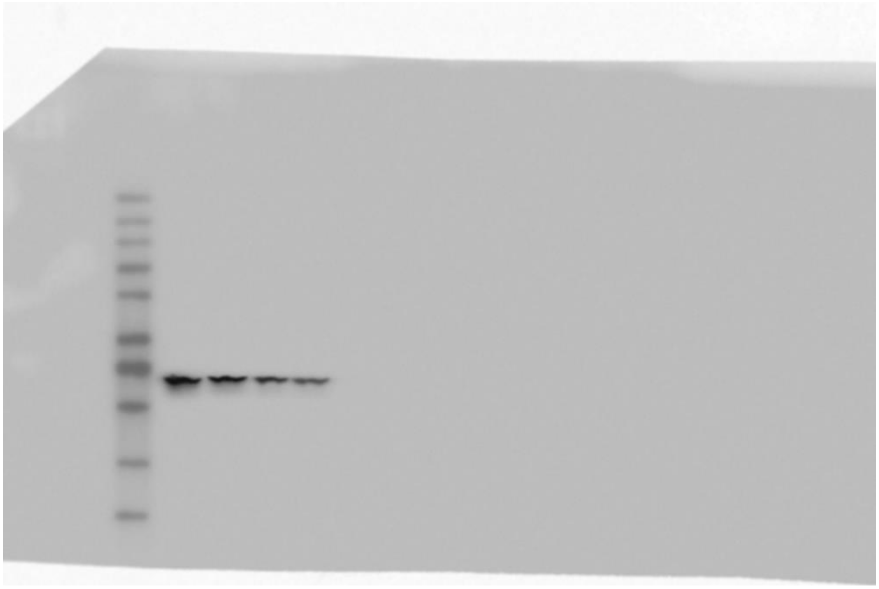

GAPDH—U343-R (Figure3)

GAPDH 36kDa-

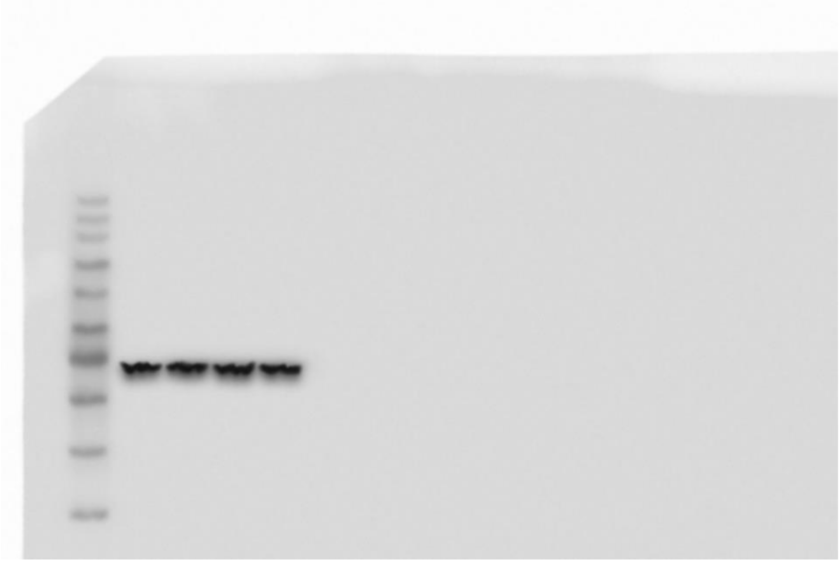

GAPDH—U251-R (Figure3)

GAPDH 36kDa-

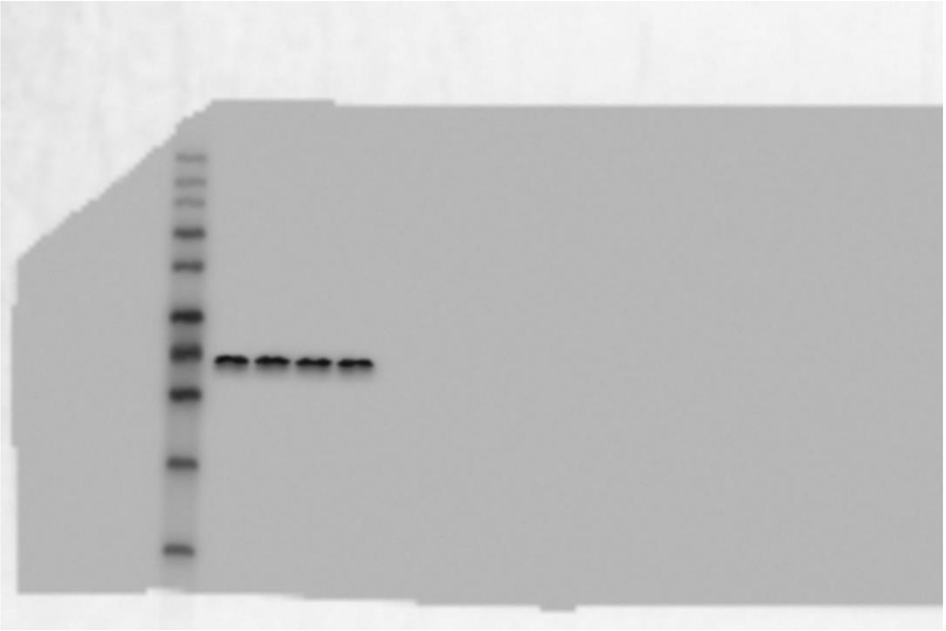

E-cadherin--U343-R (Figure3)

E-cadherin 135kDa-

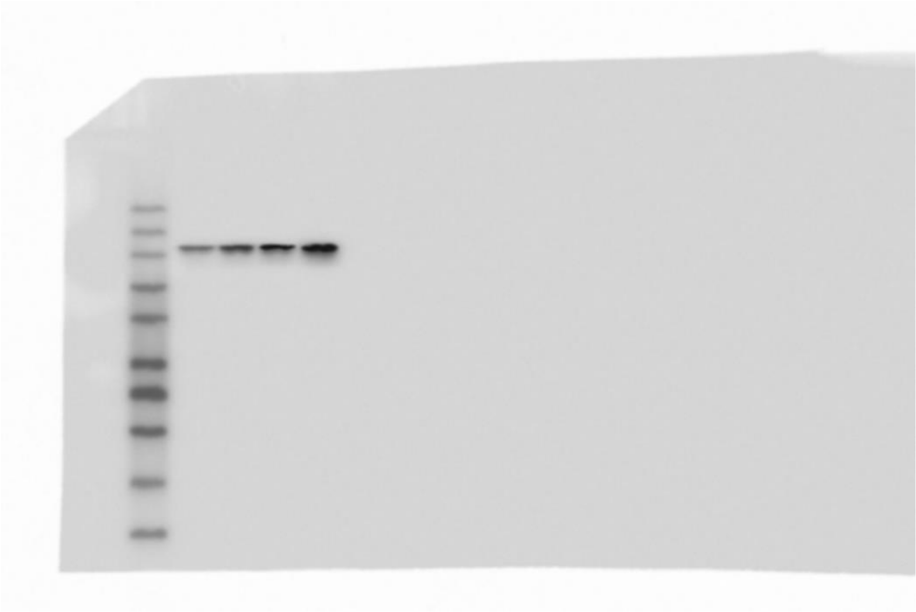

E-cadherin--U343-R (Figure3)

E-cadherin 135kDa-

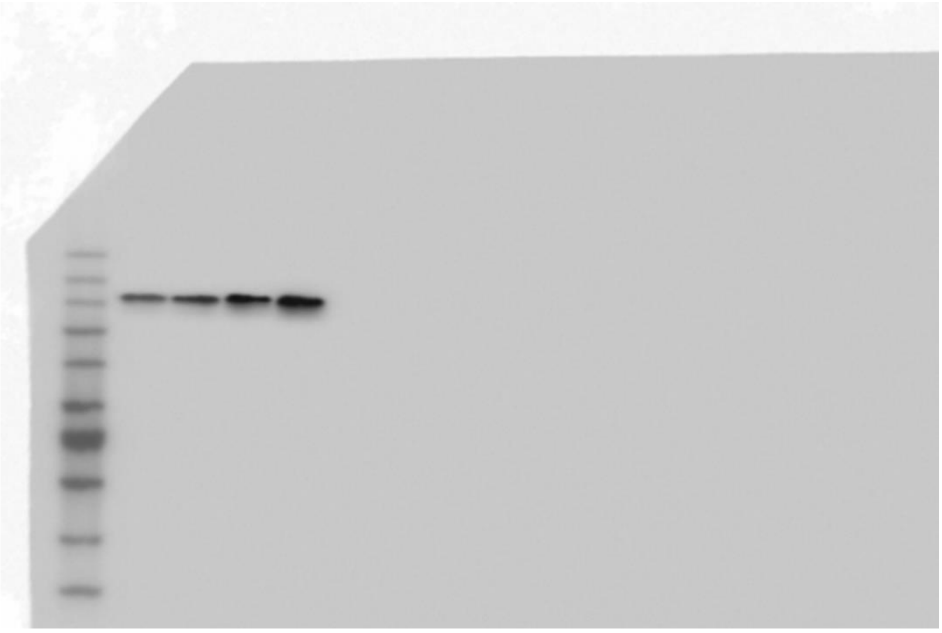

GAPDH—U343-R (Figure3)

GAPDH 36kDa-

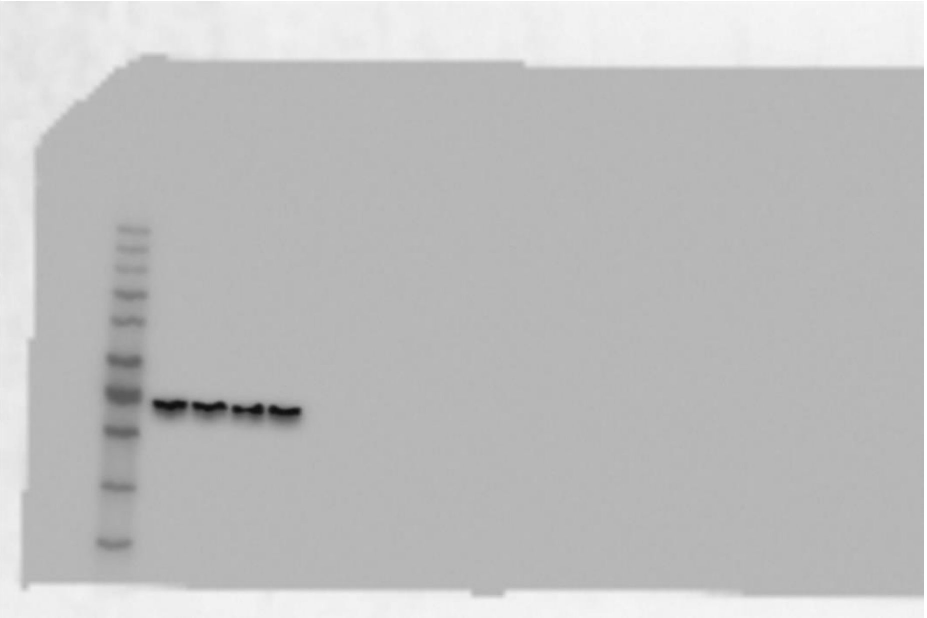

GAPDH—U251-R (Figure3)

GAPDH 36kDa-

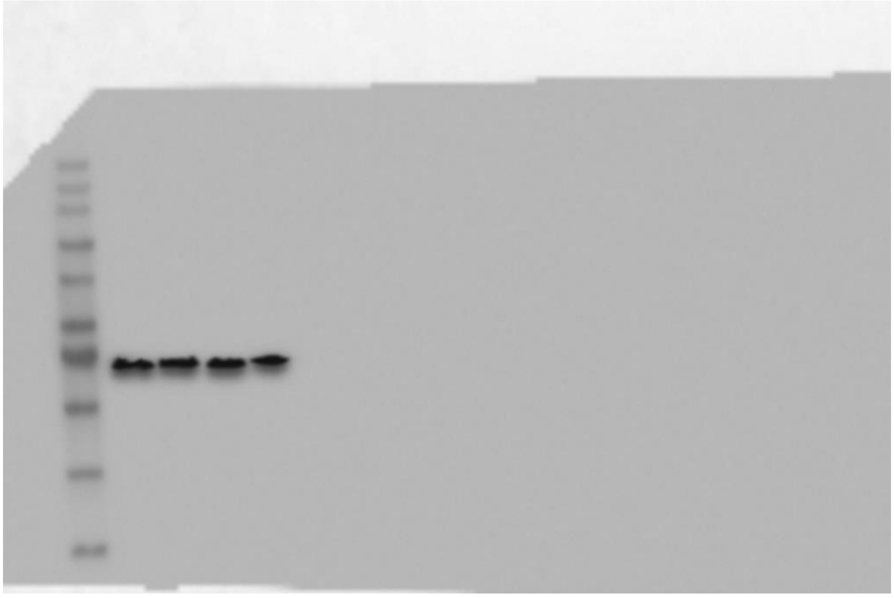

N-cadherin—U343-R (Figure3)

N-cadherin 140kDa -

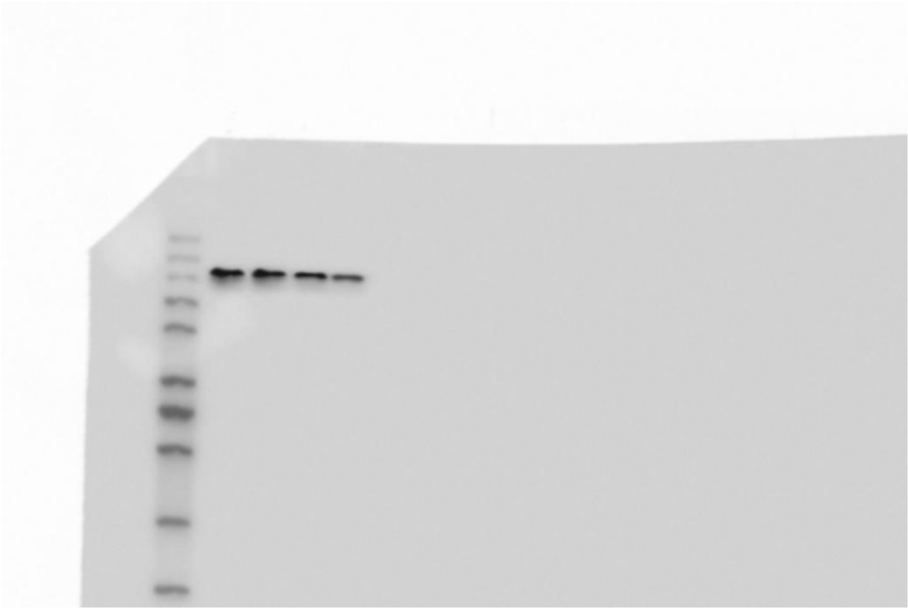

N-cadherin—U251-R (Figure3)

N-cadherin 140kDa -

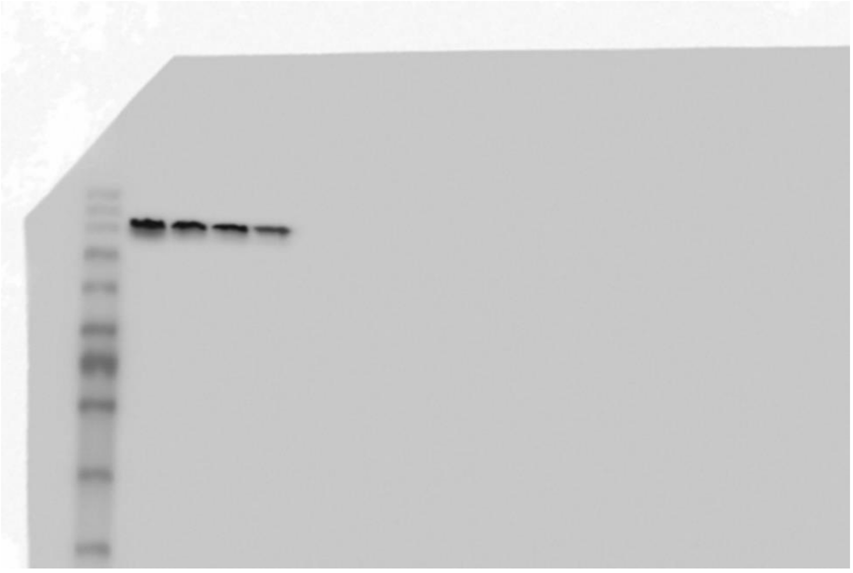

GAPDH—U343-R (Figure3)

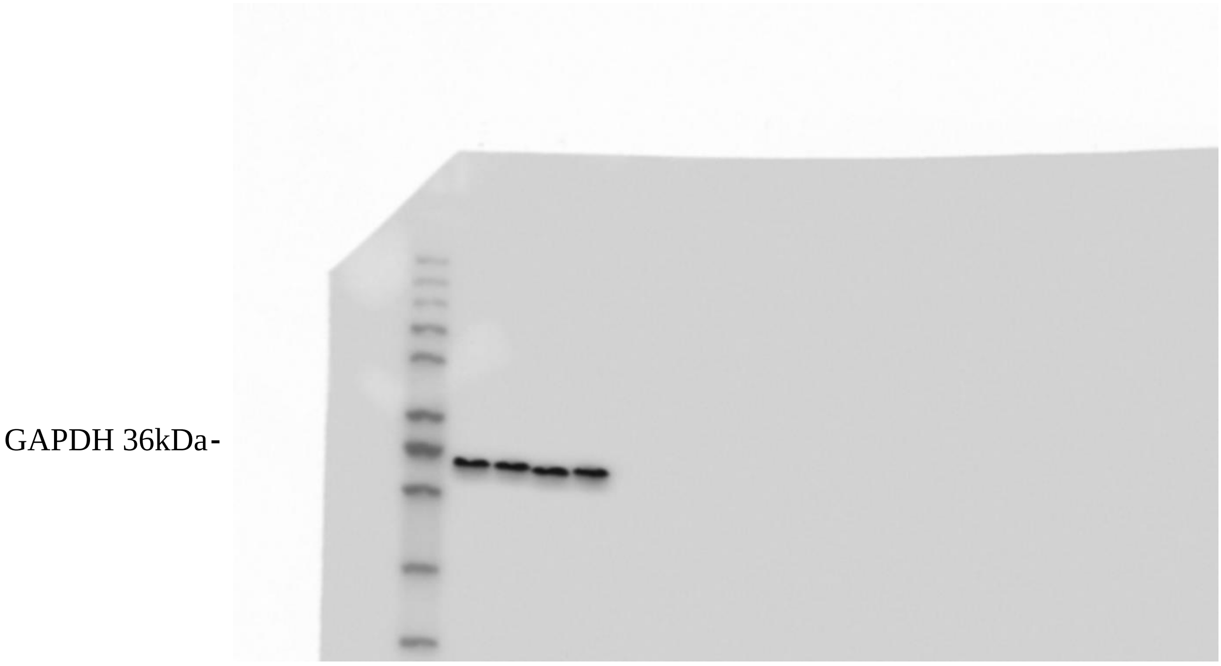

GAPDH—U251-R (Figure3)

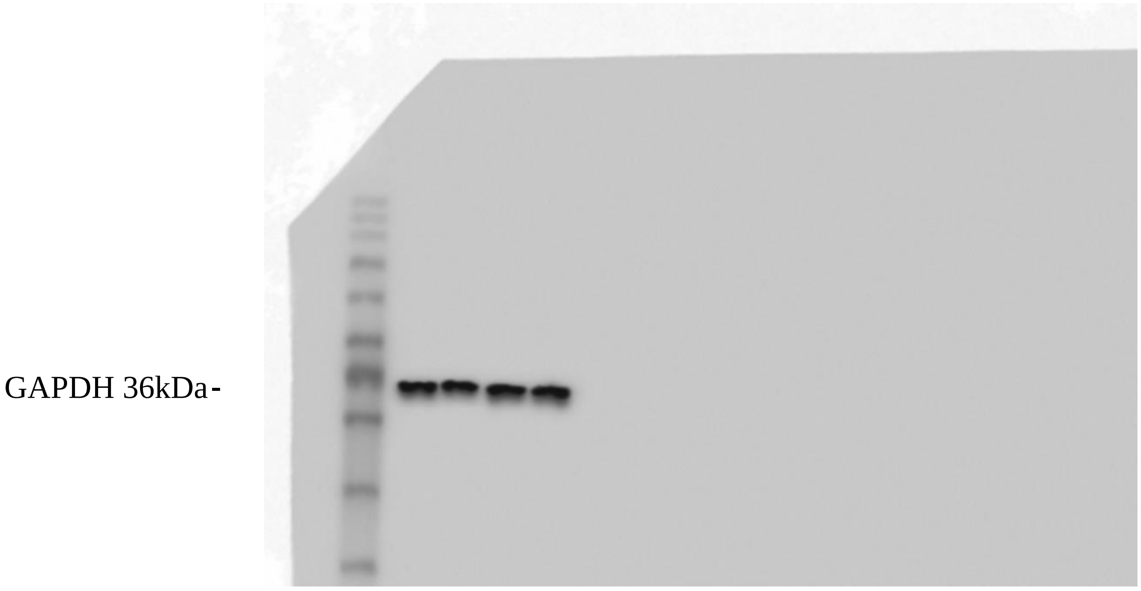

MGMT—U343-R (Figure3)

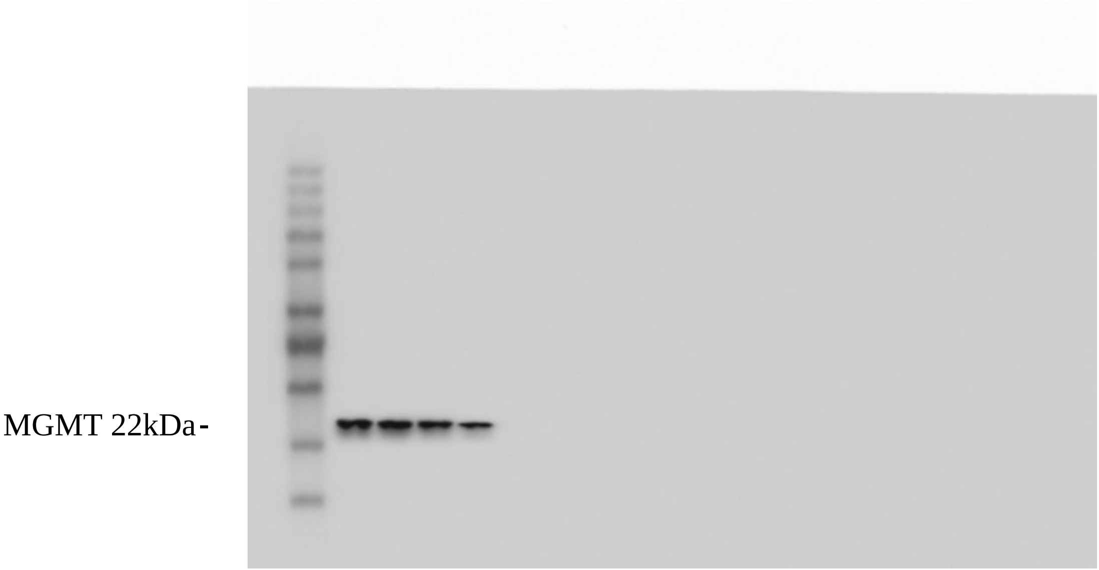

MGMT—U251-R (Figure3)

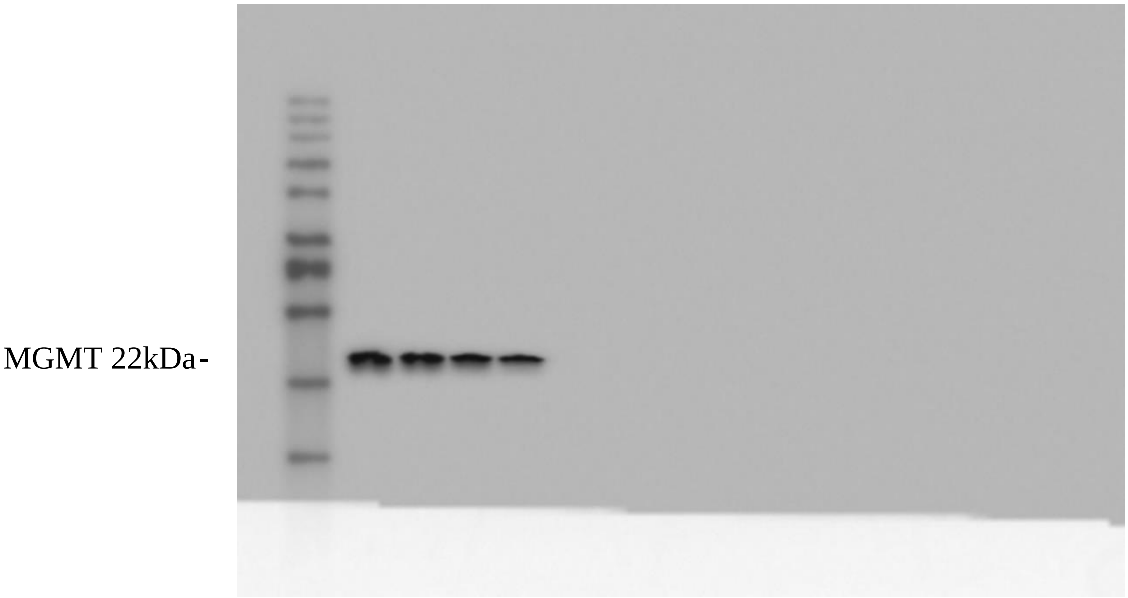

GAPDH—U343-R (Figure3)

GAPDH 36kDa-

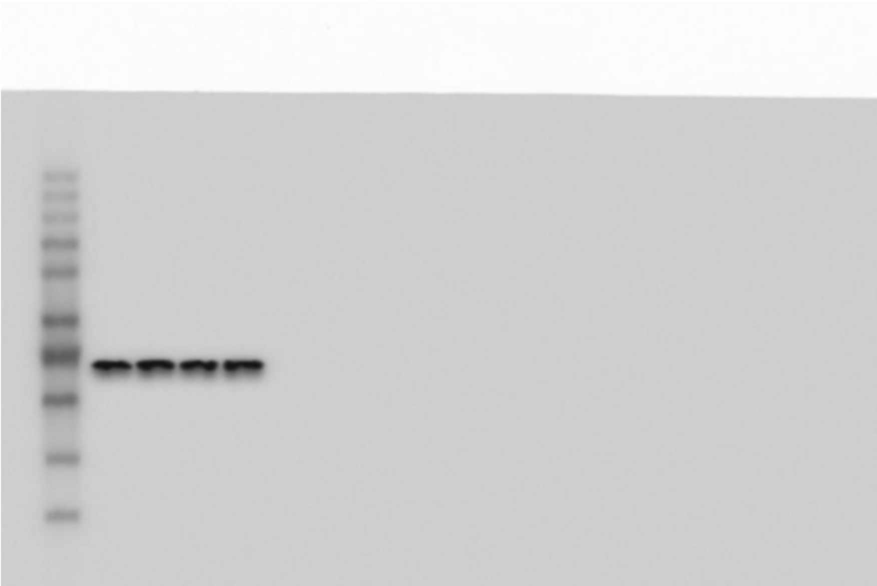

GAPDH—U251-R (Figure3)

GAPDH 36kDa-

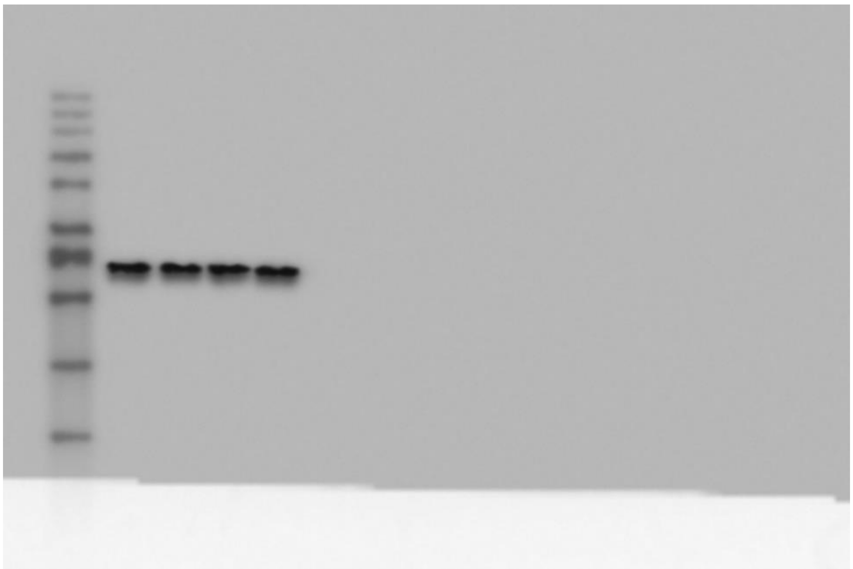

MGMT—U343-R (Figure4)

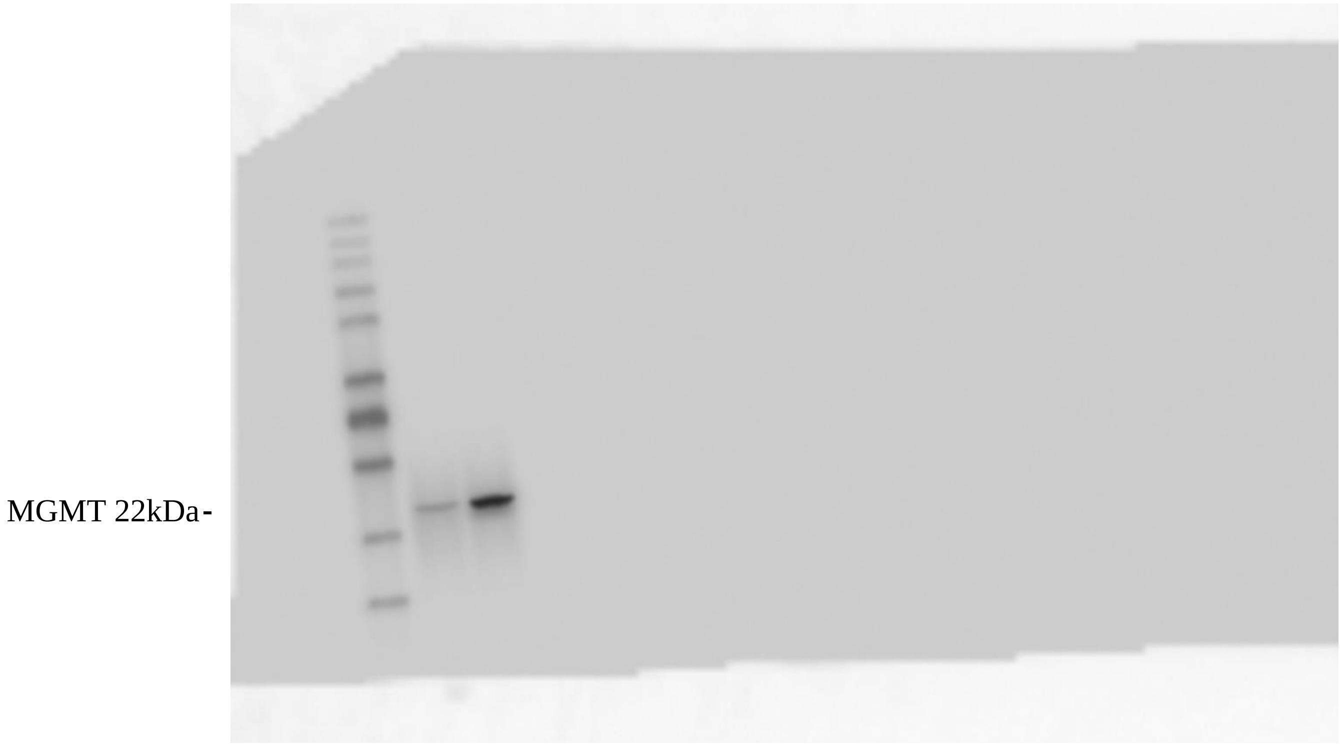

MGMT—U251-R (Figure4)

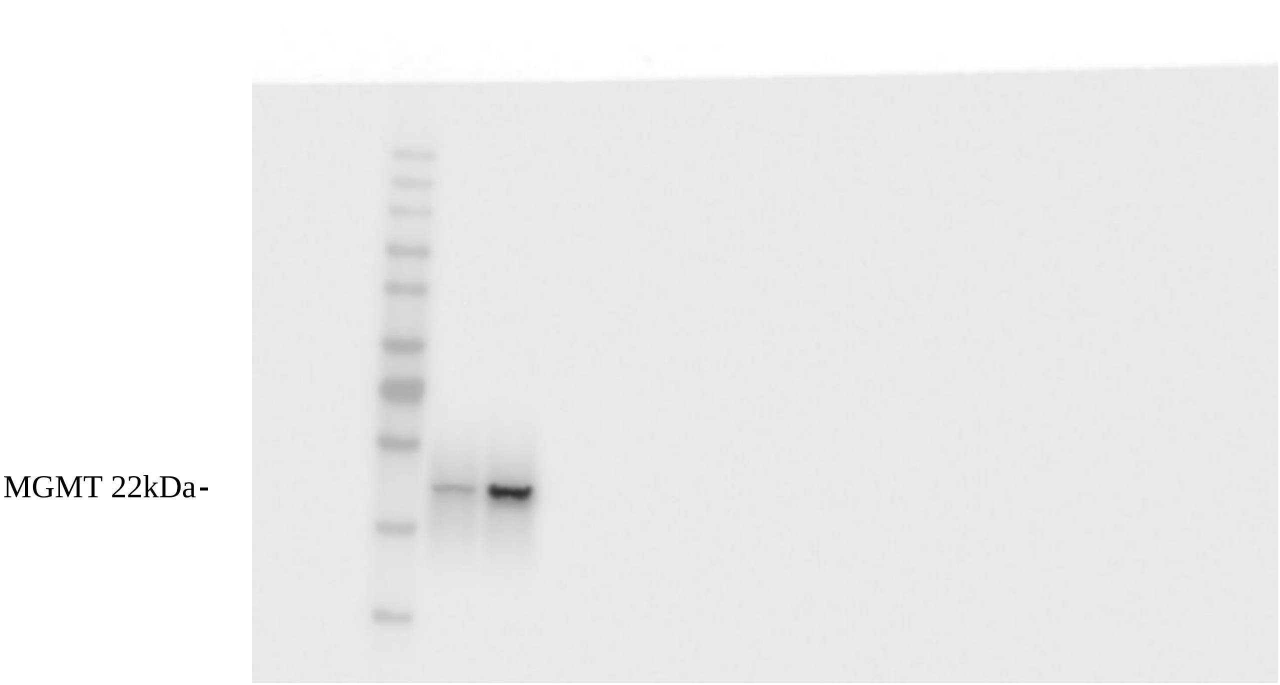

β-actin—U343-R (Figure4)

β-actin 42kDa-

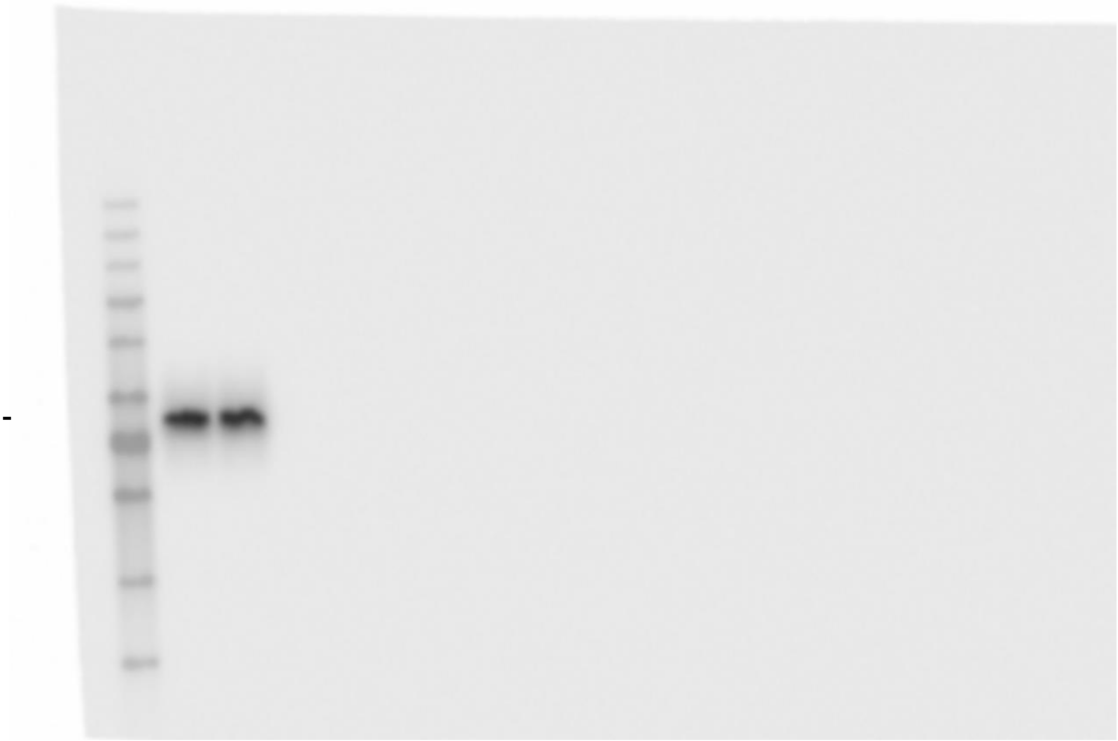

β-actin—U343-R (Figure4)

β-actin 42kDa-

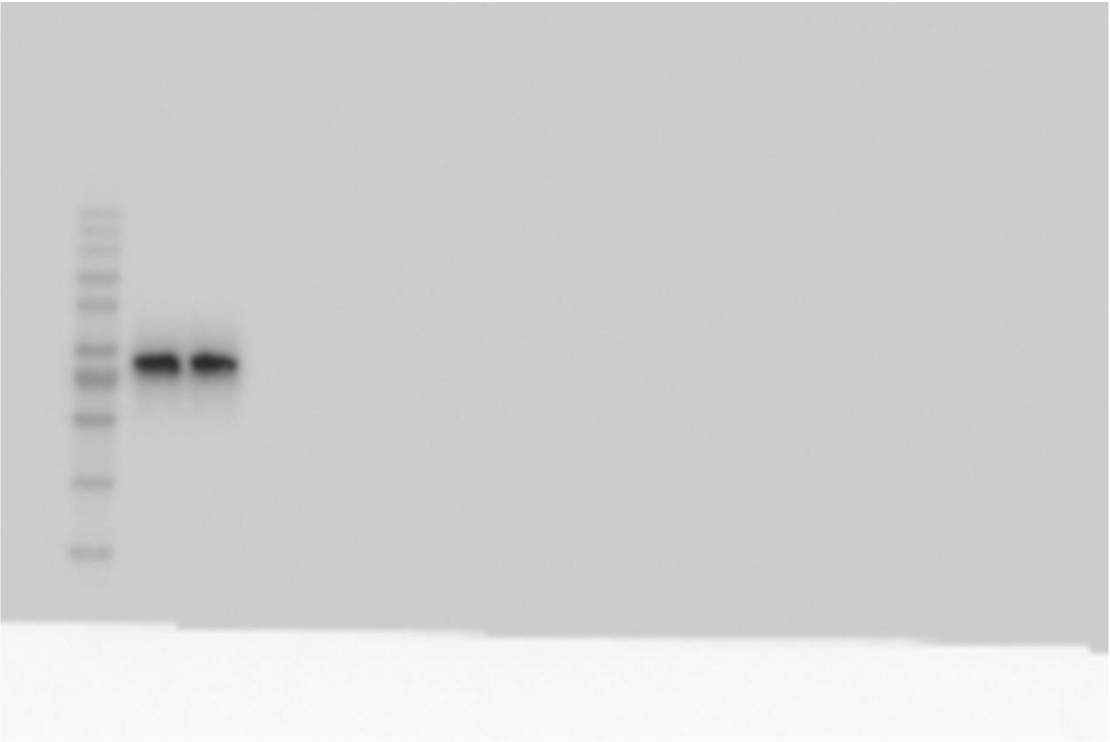

MGMT (Figure6)

MGMT 22kDa-

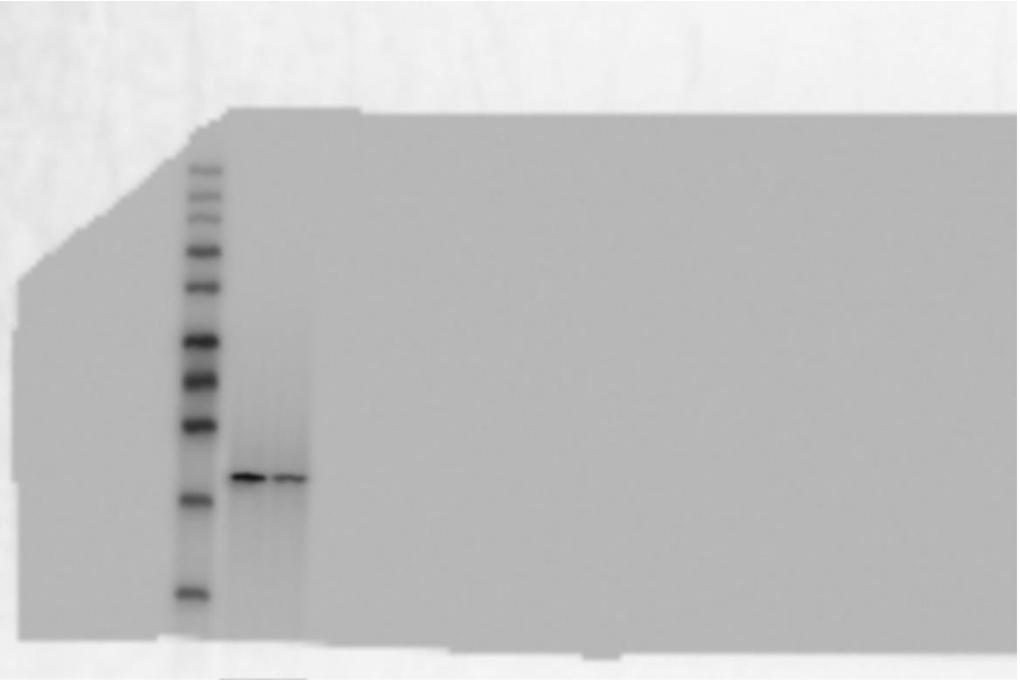

GAPDH (Figure6)

GAPDH 36kDa-

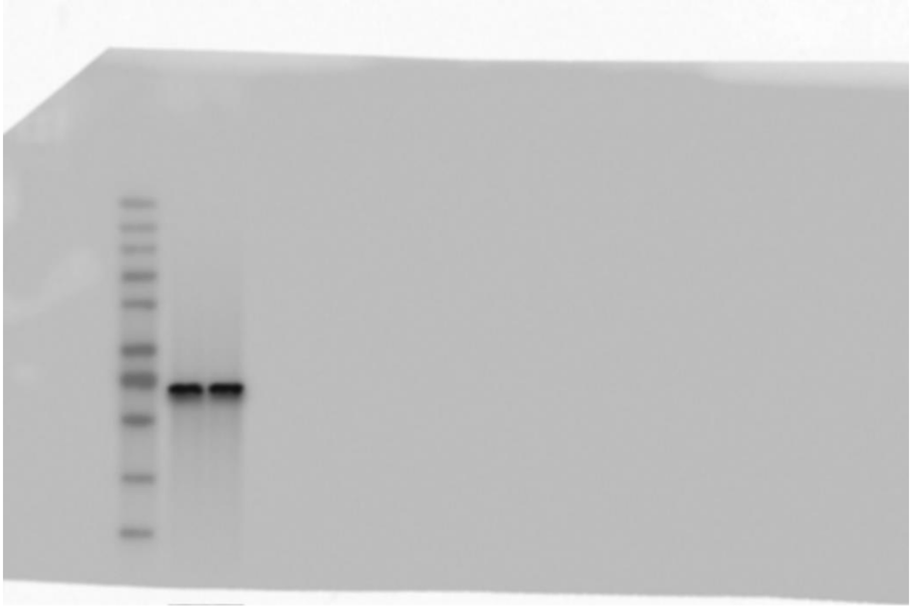

PCNA—U343-R (Figure S3)

PCNA 36kDa-

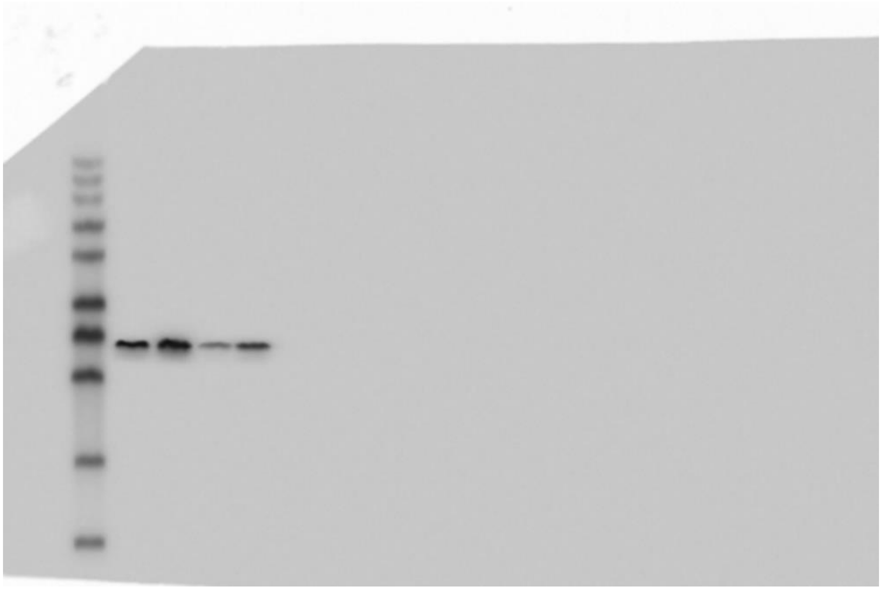

PCNA—U251-R (Figure S3)

PCNA 36kDa-

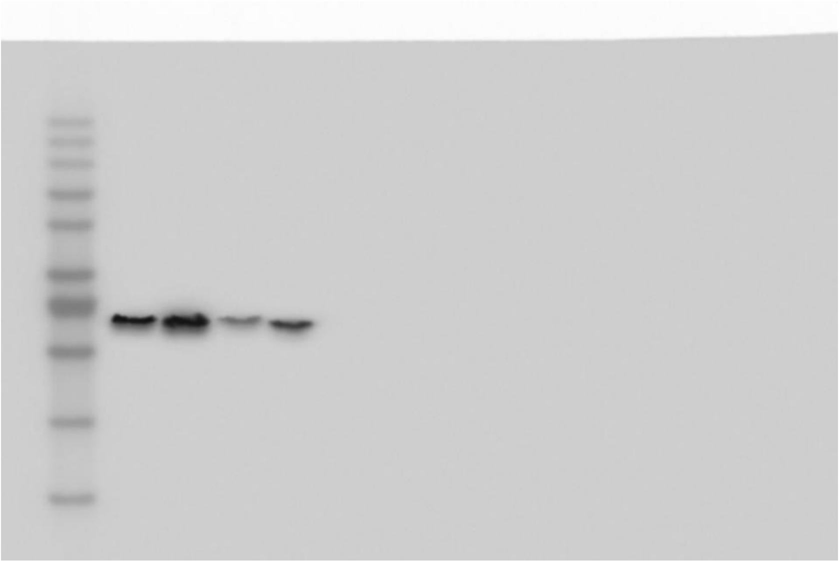

GAPDH—U343-R (Figure S3)

GAPDH 36kDa-

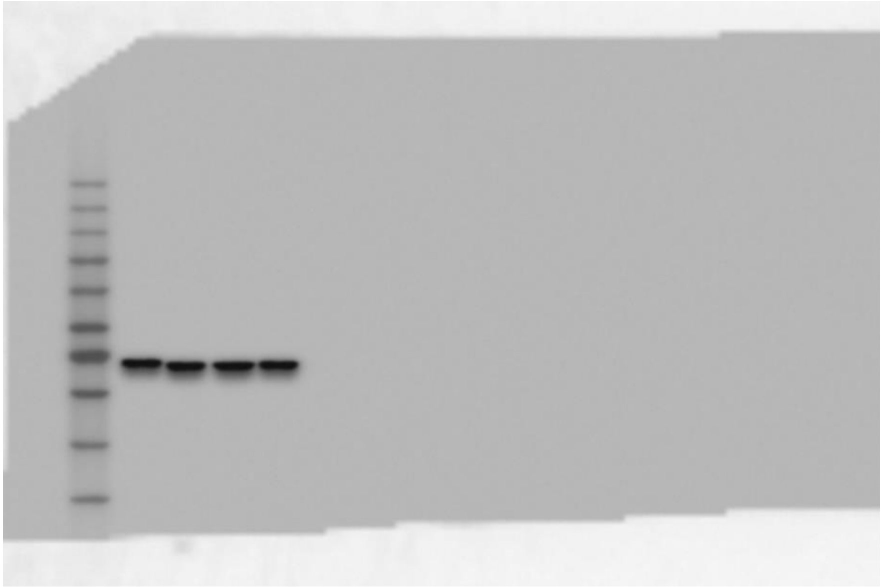

GAPDH—U251-R (Figure S3)

GAPDH 36kDa-

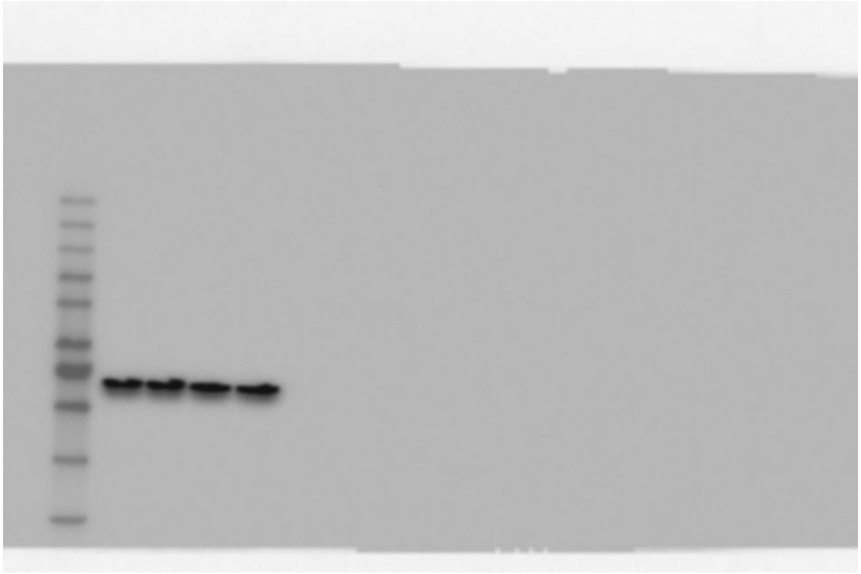

E-cadherin—U343-R (Figure S3)

E-cadherin 135kDa -

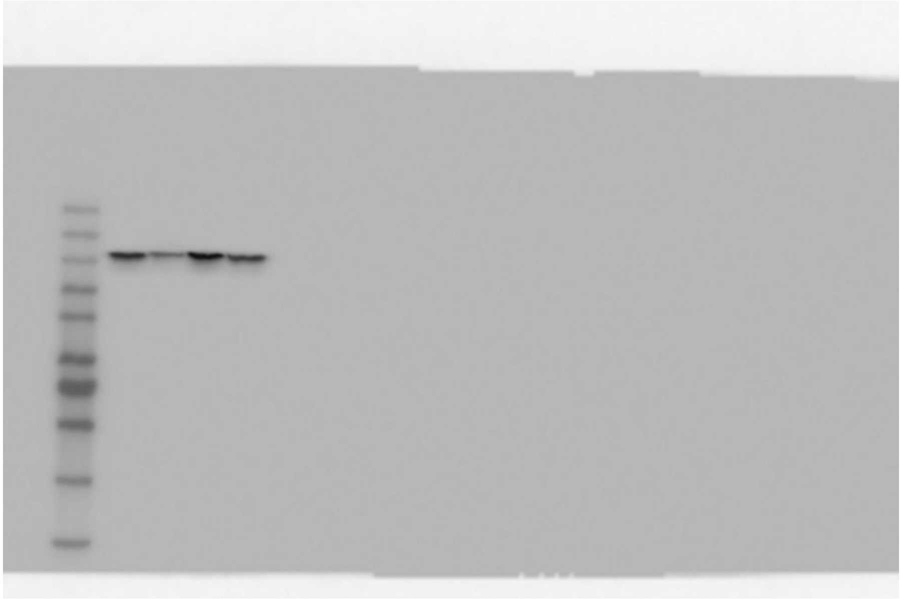

E-cadherin—U251-R (Figure S3)

E-cadherin 135kDa -

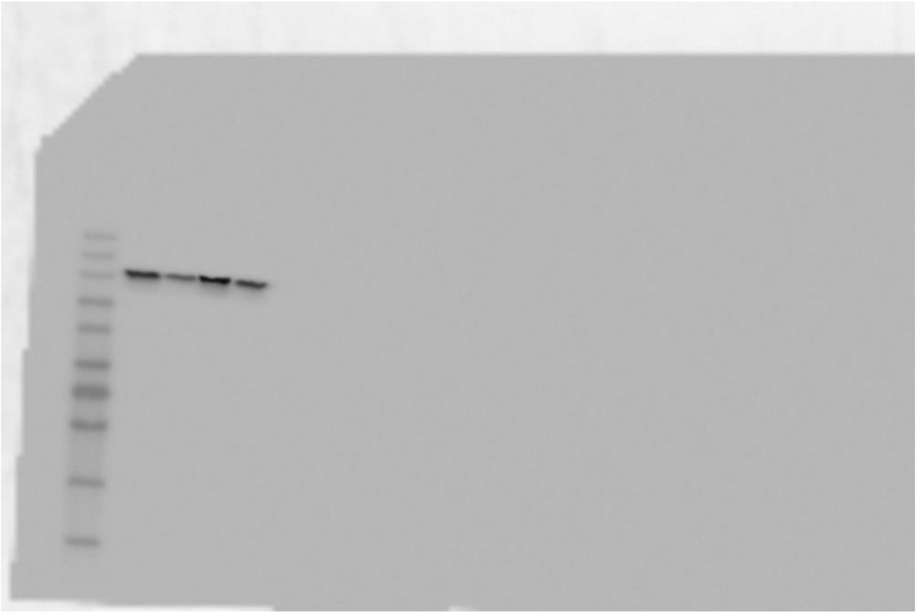

GAPDH—U343-R (Figure S3)

GAPDH 36kDa-

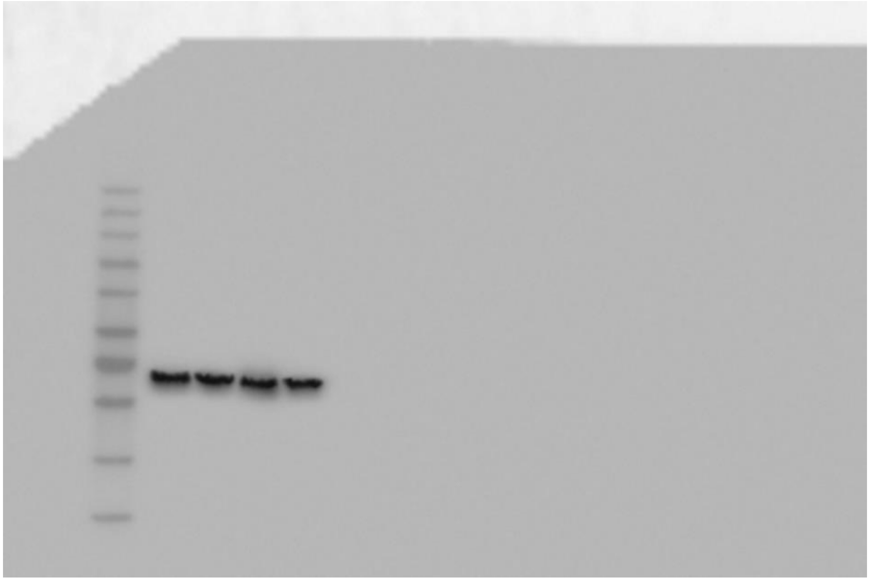

GAPDH—U251-R (Figure S3)

GAPDH 36kDa-

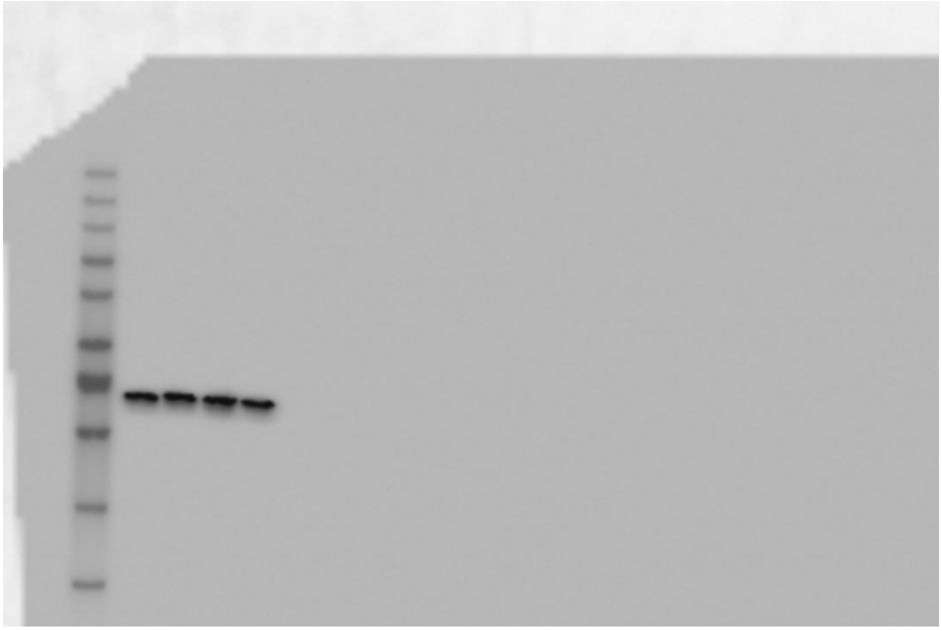

N-cadherin —U343-R (Figure S3)

N-cadherin 140kDa -

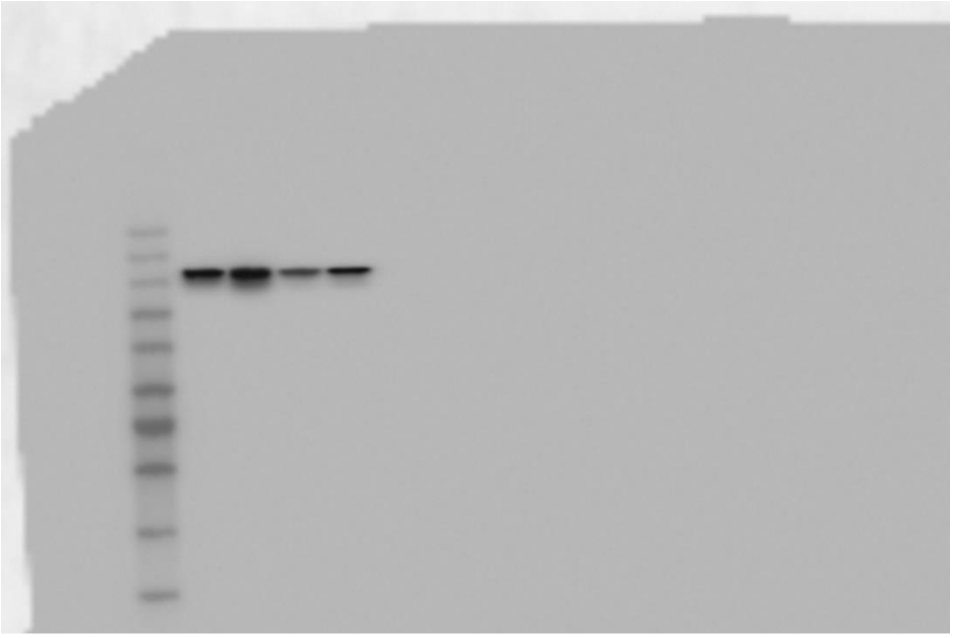

N-cadherin —U251-R (Figure S3)

N-cadherin 140kDa -

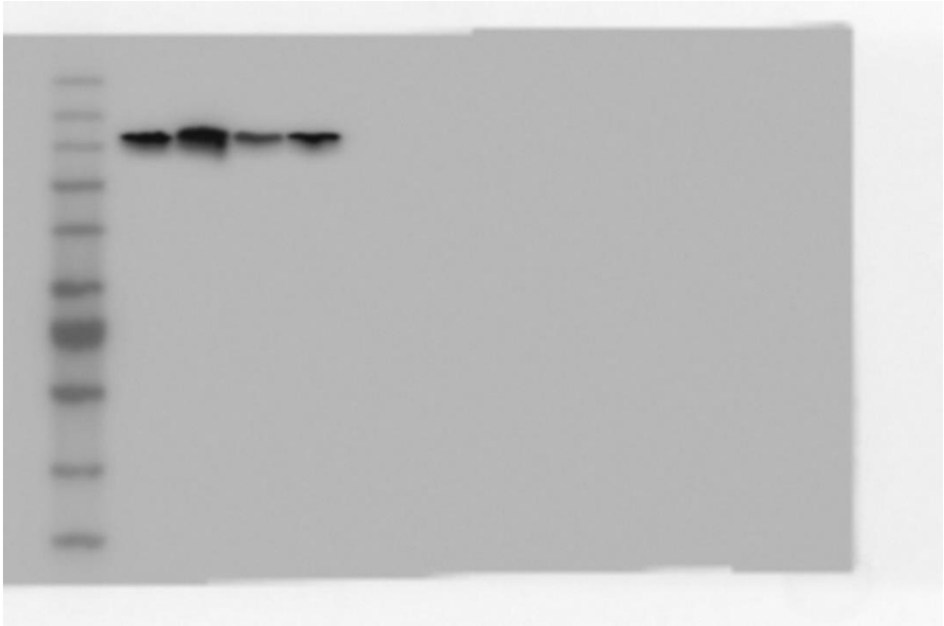

GAPDH—U343-R (Figure S3)

GAPDH 36kDa-

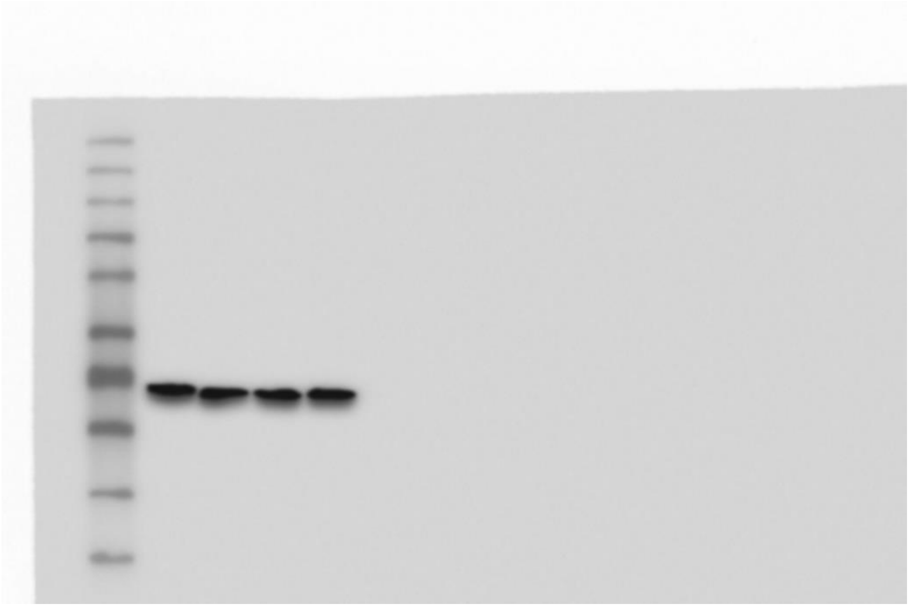

GAPDH—U251-R (Figure S3)

GAPDH 36kDa-

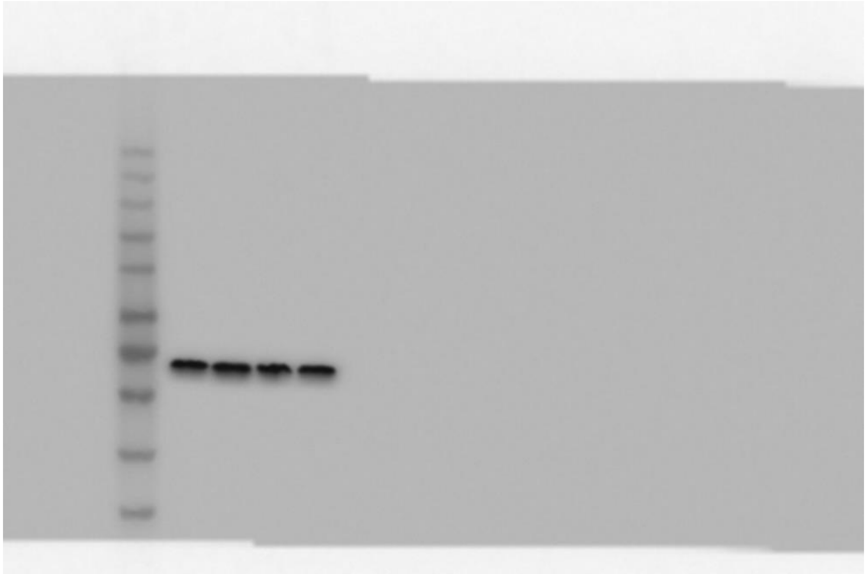

MGMT—U343-R (Figure S3)

MGMT 22kDa-

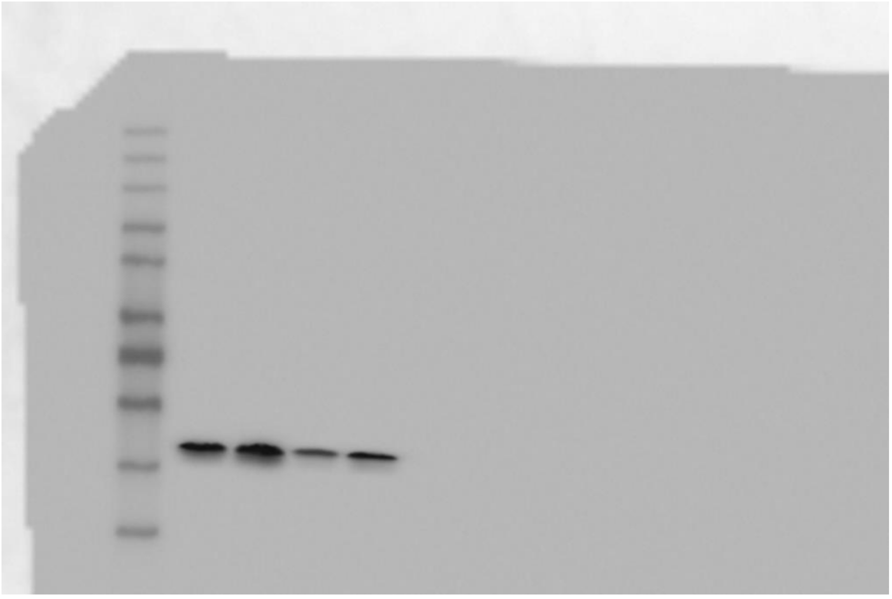

MGMT—U251-R (Figure S3)

MGMT 22kDa-

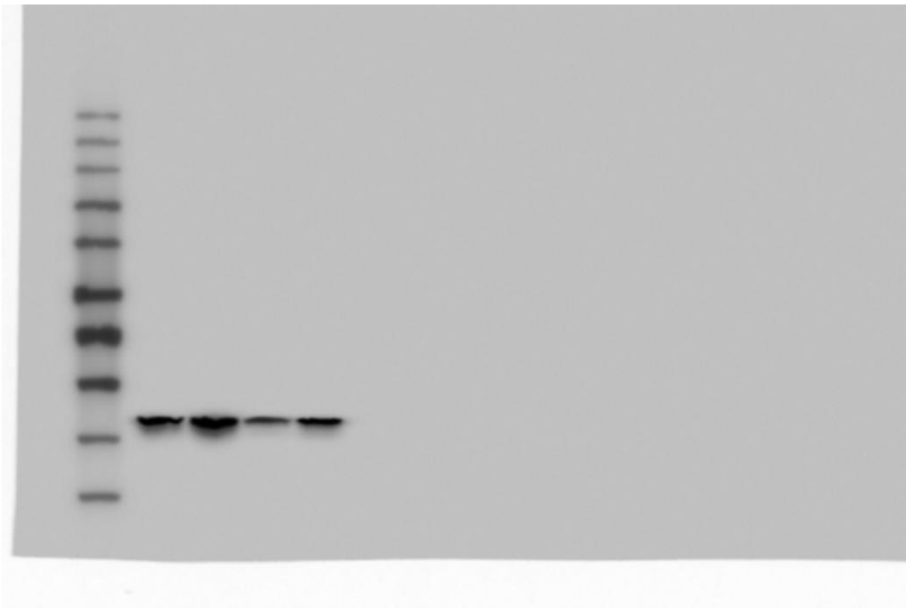

GAPDH—U343-R (Figure S3)

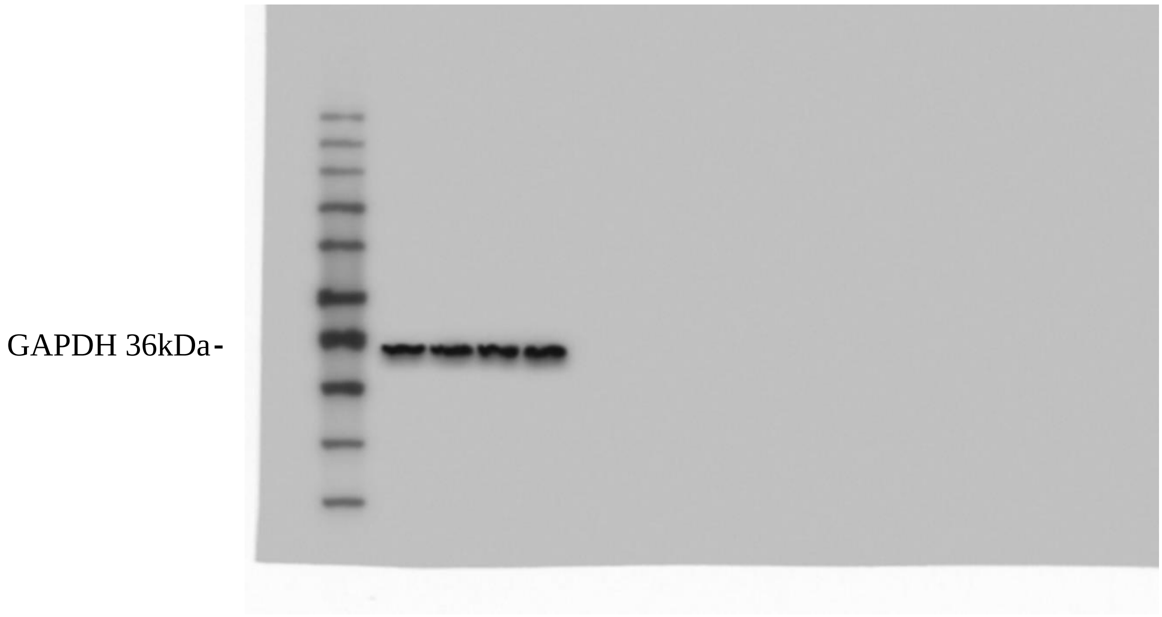

GAPDH—U251-R (Figure S3)

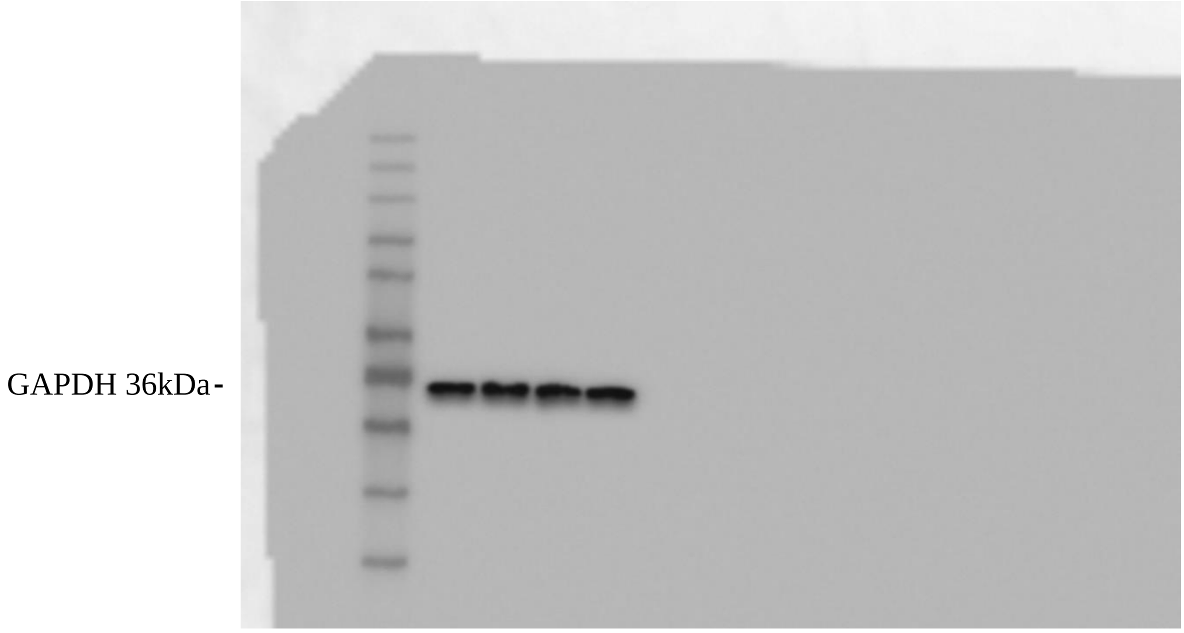

Supplement: Supplementary file 7 — Original Data File [file 41419_2022_5056_MOESM7_ESM.pdf]
